# Genome-Wide DArTSeq Genotyping and Phenotypic Based Assessment of Within and Among Accessions Diversity and Effective Sample Size in the Diverse Sorghum, Pearl Millet, and Pigeonpea Landraces

**DOI:** 10.3389/fpls.2020.587426

**Published:** 2020-12-14

**Authors:** Victor Allan, Mani Vetriventhan, Ramachandran Senthil, S. Geetha, Santosh Deshpande, Abhishek Rathore, Vinod Kumar, Prabhat Singh, Surender Reddymalla, Vânia C. R. Azevedo

**Affiliations:** ^1^Centre for Plant Breeding and Genetics, Tamil Nadu Agricultural University (TNAU), Coimbatore, India; ^2^International Crops Research Institute for the Semi-Arid Tropics (ICRISAT), Hyderabad, India

**Keywords:** DArTseq, within accession diversity, effective population size, landraces, pearl millet, pigeonpea, regeneration, sorghum

## Abstract

Germplasm should be conserved in such a way that the genetic integrity of a given accession is maintained. In most genebanks, landraces constitute a major portion of collections, wherein the extent of genetic diversity within and among landraces of crops vary depending on the extent of outcrossing and selection intensity infused by farmers. In this study, we assessed the level of diversity within and among 108 diverse landraces and wild accessions using both phenotypic and genotypic characterization. This included 36 accessions in each of sorghum, pearl millet, and pigeonpea, conserved at ICRISAT genebank. We genotyped about 15 to 25 individuals within each accession, totaling 1,980 individuals using the DArTSeq approach. This resulted in 45,249, 19,052, and 8,211 high-quality single nucleotide polymorphisms (SNPs) in pearl millet, sorghum, and pigeonpea, respectively. Sorghum had the lowest average phenotypic (0.090) and genotypic (0.135) within accession distances, while pearl millet had the highest average phenotypic (0.227) and genotypic (0.245) distances. Pigeonpea had an average of 0.203 phenotypic and 0.168 genotypic within accession distances. Analysis of molecular variance also confirms the lowest variability within accessions of sorghum (26.3%) and the highest of 80.2% in pearl millet, while an intermediate in pigeonpea (57.0%). The effective sample size required to capture maximum variability and to retain rare alleles while regeneration ranged from 47 to 101 for sorghum, 155 to 203 for pearl millet, and 77 to 89 for pigeonpea accessions. This study will support genebank curators, in understanding the dynamics of population within and among accessions, in devising appropriate germplasm conservation strategies, and aid in their utilization for crop improvement.

## Introduction

Plant genetic resources include landraces, wild and weedy relatives, improved cultivars, etc. which are of potential value as a resource for present and future generations of people. Landraces occupy a major portion in collections conserved in genebanks. Landraces possess a multifaceted evolutionary history and a vast diversity, primarily associated with humans, also influenced by both natural and farmers' informal selections (Hawkes, 1983). The high variability and genetic diversity of landraces are well-known. Harlan (1965) reported the gene-flow from weeds to landraces and several other authors (Ellstrand et al., 1999; Jarvis and Hodgkin, 1999; Messeguer, 2003; Gompert and Buerkle, 2016) reported the transfer and diffusion of genes into landraces from various sources in both self and out-crossing species. Harlan (1971) emphasized landraces as genetically dynamic populations, and a result of millennia of artificial and natural selection, also Hawkes (1983) described landraces as highly diverse populations or a mixture of heterogenous genotypes, and several other authors proposed various definitions to landraces, explaining their heterogeneity and genetic nature (Brown, 1978; Martin and Adams, 1987; Astley, 1991; Michaelis et al., 1991). Brown (1978), Bellon (2009), and Frankel and Soulé (1981) explained the occurrence of within and between population genetic variation in landrace populations and further explained the within-population diversity is mainly an effect of heterogeneity over space and time. Many pieces of literature are available emphasizing the high variability in landraces, however, only a few studies are available investigating diversity within individuals of landrace accessions that are conserved in genebanks (Busso et al., 2000; Bhattacharjee et al., 2002), while few other studies focused on diversity within landrace populations conserved *in situ* (Djè et al., 1999; Pressoir and Berthaud, 2004; Dreisigacker et al., 2005; Al Khanjari et al., 2007; Jones et al., 2008; Hagenblad et al., 2012; Kyratzis et al., 2019).

Therefore, understanding the diversity within landraces is essential to make sure that, in genebanks the genetic integrity of a given accession is maintained with its innate variability and diversity without losing any rare allele variants. The major cause for allele loss in genebank accessions is genetic drift when accessions are regenerated with small sample sizes (Crossa, 1995). Mode of pollination being the key factor governing the frequencies of alleles within different individuals of a population, it influences the variability, quantum of diversity, gene flow and population dynamics behind evolution. Hammer et al. (1996) explained the effect of mode of pollination on genetic erosion of landraces, Zeven (1998) explained the attainment of gradual homozygosity within inbreeding landrace populations, and Villa et al. (2005) explained the influence of mode of reproduction in alteration of genetic structure of landraces. Genebanks exercise various scientific strategies to preserve the inherent genetic variability within each accession with theoretical foundations of various population genetic considerations, mainly the mode of reproduction, allelic frequencies, distribution of allelic variations, the proportion of rare alleles, etc. to maintain the genetic integrity of an accession. Rare alleles, however, are easily susceptible to random genetic drifts and can be lost permanently (Ramanatha Rao and Hodgkin, 2002) when handled with inadequate scientific knowledge about the underlying population dynamics. Thus, appropriate conservation strategies with statistically estimated population sizes should be followed. In this study, we have chosen three crops that differ in pollination behavior, including highly cross pollinated pearl millet (>85%) (Burton, 1983), and often-cross pollinated sorghum (about 18%) (Barnaud et al., 2008) and pigeonpea (about 30%) (Saxena et al., 1990), to comparatively assess the within and between accession diversity. Landraces of these crops possess large variability within accessions, therefore chosen for this study.

Classical molecular markers used to assess the genetic diversity in these crops included SSR (Budak et al., 2003; Chandra-Shekara et al., 2007; Bashir et al., 2015), RFLP (Bhattacharjee et al., 2002; Govindaraj et al., 2009), ISSR (Kumar et al., 2006; Animasaun et al., 2015), RAPD (Chowdari et al., 1998; Chandra-Shekara et al., 2007), SRAP (Xie et al., 2010), etc. However, these molecular markers had constrains such as high cost of genotyping per sample and most of these technologies are gel-based and lacked the ability to rapidly analyze large number of marker loci. Recent technological developments in high throughput genotyping overcame these limitations and technologies like DArTSeq, by combining DArT (Diversity Array Technology) with NGS (Next Generation Sequencing), offered the flexibility of genome-wide characterization of germplasms, even without prior sequence information, parallelly providing a low-cost platform for high throughput marker genotyping. Several studies using DArTSeq on diversity and population structure assessments have been reported on various crops evidencing the potential scope of this technology in diversity assessment (Pailles et al., 2017; Raman et al., 2017; Barilli et al., 2018; Edet et al., 2018; Ndjiondjop et al., 2018). What makes DArTSeq to stand apart from other GBS (Genotyping By Sequencing) techniques is their method of complexity reduction that are targeted over the genomic coding regions and the additional advantage of genotyping without prior sequence information extents its scope even toward the under researched wild accessions. It also offers relatively better genome coverage with high reproducibility as DArTSeq is performed at higher sequencing depths and uses strict filtering criterions, it generates markers with less missing data compared to other GBS approaches.

With these background, this study aims (i) to assess genotypic (DArTSeq) and phenotypic characterization of geographically representative diverse sorghum, pearl millet, and pigeonpea landraces and wild accessions to comparatively investigate the extent of diversity within and among accessions, and (ii) to assess the minimum sample (population) size required to capture 95% of the alleles with an expected probability of 95%, from the least frequent allele or from the frequency of the rarest allele for each accession. The scope of this study aims to benefit genebank curators in understanding the dynamics of population within and among accessions, and devising proper sampling strategies (sample size) while regeneration, for effective genebank management and for their utilization in crop improvement. To the best of our knowledge, this study is the first of its kind, and no studies were found utilizing NGS for investigating within accession diversity and sample size estimations, particularly for sorghum, pigeonpea, and pearl millet.

## Materials and Methods

### Plant Material

**Figure 1:**
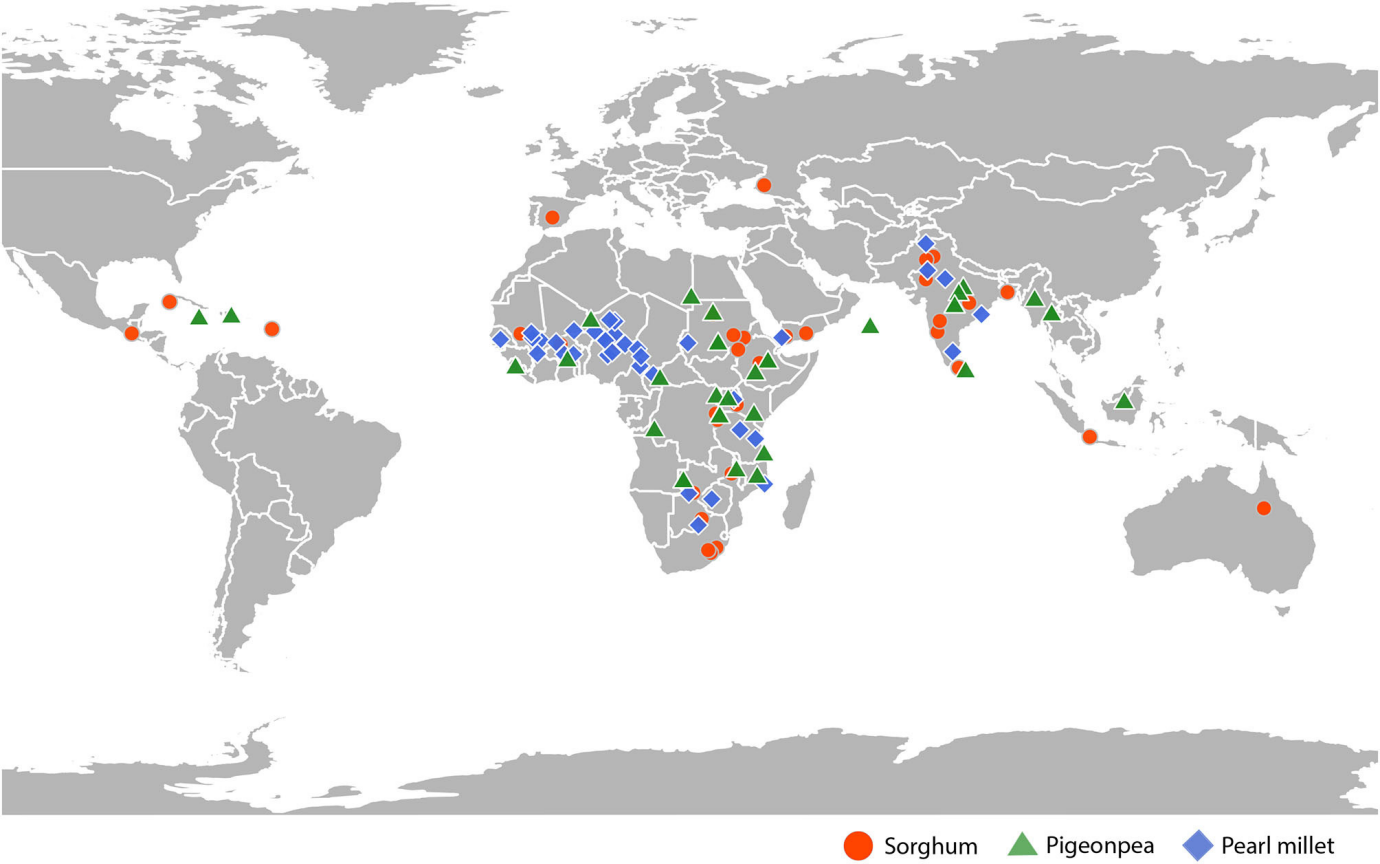
Geographical distribution of the sorghum, pigeonpea, and pearl millet accessions used in this study over continents.

This study investigated a total of 108 geographically diverse accessions of sorghum, pearl millet, and pigeonpea (Supplementary Tables 1–3) (Figure 1), conserved at ICRISAT genebank. Accessions of sorghum included 31 landraces and 5 wild accessions, collected from 26 different countries from 5 different continents, consisted of all the 5 basic races and all 10 intermediate races as classified by Harlan and de Wet (1972). Accessions of pearl millet consisted of 33 landraces and 3 wild accessions, collected from 19 different countries from 2 different continents, and accessions of pigeonpea included 36 landraces collected from 34 different countries from 5 different continents. All these 108 accessions were raised in fields during post-rainy 2018 at ICRISAT, Hyderabad, for phenotypic and genotypic characterization. Sorghum accessions were sown on black soil, whereas pearl millet and pigeonpea were sown on red soil. Accessions of sorghum occupied three-rows of 9 m length, spaced 75 cm between rows, with a plant-to-plant spacing of about 10 cm. Accessions of pearl millet were laid in 4-meter rows, with each accession occupying 4 rows, spaced 75 cm between rows and 10 cm between plants. Each accession of pigeonpea occupied two rows of 9-meter length, spaced ~75 cm between rows and 50 cm between plants.

### DNA Extraction, Complexity Reduction and Genotyping

Individual plants within each accession of sorghum, pearl millet, and pigeonpea were labeled with unique plant ID, and leaf samples were collected from 15 plants in each accession of sorghum and pigeonpea, and 25 plants from each accession of pearl millet, totaling a 540, 900 and 540 samples in sorghum, pearl millet, and pigeonpea respectively. Leaf samples were collected from 15 days old seedlings of sorghum and pearl millet, and 2-month old seedlings of pigeonpea. Collected leaf samples were sealed in zip lock bags or collected using the PCR plates with corresponding plant ID for each sample and packed with ice cubes, and sent for DNA extraction on the same day. The DNA extraction was carried out following the procedure reported by Mace et al. (2003) and the extracted genomic DNA samples were sent to DArT Private Limited in Canberra, Australia (www.diversityarrays.com) for DArTSeq genotyping.

### SNP Filtering

The SNP markers from DArTSeq were filtered with a maximum threshold of 95% reproducibility, 80% call rate for markers, and 50% missing values over samples. The SNPs were not filtered for minor allele frequencies (MAF) in order to preserve the rare allele variants, which have the main part of the focus in this study.

### Phenotypic Evaluation

To capture maximum phenotypic variability, all the individual plants within each accession were labeled with unique plant ID and data on both qualitative and quantitative traits (Supplementary Table 4) were recorded for all the 3 crops following the respective crop descriptors (IBPGR and ICRISAT, 1993a,b,c), throughout the growing season. In this study, a large number of plants including those plants that were used for DArTSeq and also plants that were not sampled for DArTSeq were phenotyped. The total plant count for each accession ranged from 115 to 234 in sorghum, 51 to 116 in pearl millet. However, in pigeonpea, only 35 accessions had plant count over 10. Two accessions of pigeonpea had a plant count of <14, and remaining accessions had plant counts between 21 and 33. Thus, only data from the 35 accessions of pigeonpea was used for phenotypic analysis.

### Phenotypic Data Analysis

Descriptive statistics such as mean, standard deviation, and standard error were computed for quantitative traits to assess the spread and distribution of the data. Preliminary analysis of phenotypic data included investigating diversity among accessions using the mean and range values. This was followed by *post-hoc* tests, which included Student Newman Keuls test (Newman, 1939; Keuls, 1952) and Levene's test (Levene, 1960) to verify statistical significance between means and homogeneity of variances, respectively. Gower distance metric (Gower, 1971) was used for within accession diversity assessment using both quantitative and qualitative data. Pairwise distances between individual plants were subjected to the ward.D2 agglomerative clustering algorithm (Murtagh and Legendre, 2014) with 100 bootstraps. The same set of analyses were applied to all three crops. R software v.3.6.0 (R Core Team, 2019) was used with R-CRAN packages like “*cluster*” (Maechler et al., 2019) for Gower's distance computation, “*fpc*” (Hennig, 2020) and “*pvclust*” (Suzuki et al., 2019) for bootstrapped clustering, “*car*” (Fox and Weisberg, 2019) and “*agricolae*” (de Mendiburu, 2013) for SNK test and Levene's test, respectively.

### Genotypic Data Analysis

DArTSeq derived SNP data after filtering were used for analysis. Analysis of Molecular Variance (AMOVA) was computed as proposed by Excoffier et al. (1992), which partitioned the total variance into within and among population variance components. AMOVA was carried out considering each accession (with 15 or 25 individuals) as a separate population. For testing the significance, results of AMOVA were subjected to Monte Carlo's estimate of *p*-values with 99 permutations. Heterozygosity was estimated as reported by Nei (1973). For diversity assessment, Euclidean based modified Roger's distance metric (Goodman and Stuber, 1983) was used and distances between individual plants were computed, which was followed by ward.D2 agglomerative clustering (Murtagh and Legendre, 2014) and a dendrogram was produced. The “*clusterboot*” function from the R-package “*fpc*” (Hennig, 2020) and the “*aboot*” function from the R-package “*poppr*” (Kamvar et al., 2015) were used to evaluate the clusters with 100 bootstraps. Shannon diversity (*H*′) (Shannon, 1948) was calculated for each accession using the formula,

$$\begin{array}{l} H^{\prime }=-\overset{A}{\underset{a=1}{\sum }}p_{i}\log_{2}\left(p_{i}\right) \end{array}$$

Where *p*_*i*_ is the estimated frequency of the allele “*a*” on the whole sample and A is the total number of alleles in the sample.

Population structure was assessed by DAPC (Discriminant Analysis of Principle Components) using posterior membership probabilities while assessing the membership stability by estimation of *a-scores*. Phenotypic and genotypic distance matrices were subjected to Mantel's correlation with permutation tests (Mantel, 1967). The minimum seed sample size required to capture 95% of alleles within an accession with a 95% certainty, during sampling for regeneration, was calculated as reported by Crossa (1989) for each accession. Considering the rarest biallelic locus (SNP), two alleles *B*_1_ and *B*_2_with frequencies of *p*_1_and *p*_2_, so that (*p*_1_ + *p*_2_ = 1), the two possible outcomes will be,

$$\begin{array}{l} k_{1}=B_{1\;}\text{is not represented in the sample of }n\text{ gametes}\\ k_{2}=B_{2\;}\text{is not represented in the sample of }n\text{ gametes} \end{array}$$

Thus the probability of getting at least one copy of the each *B*_1_ and *B*_2_will be $P\left(k_{1}^{c}{\cap k}_{2}^{c}\;\right)$,

$$\begin{array}{l} P\left(k_{1}^{c}{\cap k}_{2}^{c}\right)=1-{\left(1-p_{1}\right)}^{n}-{\left(1-p_{2}\right)}^{n} \end{array}$$

All the above-mentioned analyses were performed using R software v.3.6.0 (R Core Team, 2019). Custom scripted codes were used for filtering, distance matrix, heterozygosity estimations, and seed sample size computations, also packages from R- CRAN and GitHub like “*adegenet*” (Jombart, 2008) and “*ade4*” (Dray et al., 2007) were used for computation of AMOVA and Mantel's test, respectively.

## Results

### Phenotyping

#### Descriptive Statistics and *post-hoc* Tests

The variations in the mean and range estimates indicated considerable variability among landraces and wild accessions of sorghum, pearl millet and pigeonpea. The SNK test indicated significant (*p* ≤ 0.05) mean differences among accessions (Supplementary Table 5). Levene's test indicated heterogeneous variances for all the quantitative traits in sorghum, pearl millet, and pigeonpea (Supplementary Table 6).

### Phenotypic Diversity: Within and Between Accessions

The Gower's phenotypic distance matrix (Gower, 1971) was computed to obtain pairwise distances between plants of all the accessions. Within accession distances varied from 0.038 to 0.141, 0.145 to 0.271, and 0.071 to 0.410 for sorghum, pearl millet, and pigeonpea, respectively (Table 1). In sorghum IS 13215 (0.141) had the maximum mean within accession distance followed by IS 31637 (0.136) and IS 27325 (0.136), and the accession IS 12919 (0.038) showed the lowest within accession distance followed by IS 13065 (0.046) and IS 2134 (0.048). In pearl millet, IP 12138 (0.271) showed the maximum within accession distance followed by the accessions IP 13112 (0.270) and IP 8761 (0.268), whereas the accession IP 21640 (0.145) had the lowest within accession distance followed by IP 22039 (0.159) and IP 21752 (0.162). In pigeonpea, ICP 13545 (0.410) showed the highest mean within accession distance followed by ICP 12840 (0.317) and ICP 10889 (0.299), whereas the least was noticed in ICP 7035 (0.071) followed by ICP 11485 (0.092) and ICP 9124 (0.094). The wild accessions of pearl millet had the minimum within accession distance [IP 21640 (0.145), IP 22039 (0.159), and IP 21752 (0.162)], in comparison to the overall scale of mean distance values of landraces (0.194–0.271). The same scenario was observed in sorghum where the wild accessions IS 14485 (0.065), IS 10897 (0.092), IS 11005 (0.077), IS 18833 (0.087), and IS 22428 (0.076) had low phenotypic within accession distances in comparison to the overall range of within accession distance values of landraces (0.038–0.141). On an average, distance among accessions was found to be higher than that of within accessions distance in all the three crops. Between accessions distance values were higher in accessions of sorghum (mean 0.387; range 0.308–0.415), followed by pigeonpea (mean 0.302; range 0.252–0.388), while low in pearl millet (mean 0.271; range 0.245–0.310) (Table 1).

**Table 1:** Mean phenotypic distances within and between accessions of sorghum, pigeonpea, and pearl millet.

**Sorghum**			**Pigeonpea**			**Pearl millet**		
**Accession number**	**Within accession distances**	**Distance from other accessions**	**Accession number**	**Within accession distances**	**Distance from other accessions**	**Accession number**	**Within accession distances**	**Distance from other accessions**
IS 12919	0.038	0.387	ICP 7035	0.071	0.351	IP 21640	0.145	0.302
IS 13065	0.046	0.318	ICP 11485	0.092	0.335	IP 22039	0.159	0.289
IS 2134	0.048	0.349	ICP 9124	0.094	0.314	IP 21752	0.162	0.317
IS 22407	0.050	0.335	ICP 9150	0.107	0.388	IP 6434	0.194	0.249
IS 22606	0.055	0.402	ICP 14059	0.120	0.316	IP 9446	0.205	0.259
IS 2348	0.060	0.364	ICP 13828	0.125	0.292	IP 11577	0.206	0.256
IS 14485	0.065	0.368	ICP 13628	0.132	0.252	IP 3616	0.207	0.247
IS 33844	0.069	0.342	ICP 11480	0.134	0.293	IP 13459	0.207	0.249
IS 32263	0.070	0.325	ICP 9877	0.143	0.285	IP 3389	0.207	0.245
IS 29605	0.073	0.318	ICP 7057	0.158	0.298	IP 5900	0.210	0.249
IS 13068	0.076	0.323	ICP 14296	0.179	0.263	IP 9824	0.212	0.269
IS 22428	0.076	0.346	ICP 13415	0.180	0.294	IP 10085	0.218	0.255
IS 35474	0.077	0.308	ICP 11491	0.181	0.275	IP 5441	0.218	0.257
IS 11005	0.077	0.365	ICP 9122	0.181	0.284	IP 11984	0.221	0.268
IS 18833	0.087	0.390	ICP 13575	0.189	0.269	IP 18147	0.223	0.269
IS 12965	0.088	0.415	ICP 13889	0.204	0.285	IP 17632	0.225	0.266
IS 34283	0.092	0.353	ICP 13316	0.205	0.305	IP 5253	0.228	0.293
IS 10897	0.092	0.350	ICP 6399	0.206	0.271	IP 6109	0.229	0.259
IS 14010	0.094	0.363	ICP 12190	0.212	0.277	IP 6244	0.234	0.258
IS 40238	0.096	0.347	ICP 11475	0.219	0.320	IP 4952	0.236	0.262
IS 18234	0.096	0.345	ICP 2309	0.227	0.314	IP 18157	0.236	0.260
IS 25476	0.097	0.354	ICP 14388	0.227	0.311	IP 19434	0.237	0.263
IS 3399	0.099	0.345	ICP 13546	0.228	0.284	IP 11677	0.238	0.274
IS 40031	0.099	0.357	ICP 12189	0.229	0.277	IP 20349	0.243	0.268
IS 35217	0.100	0.359	ICP 12041	0.235	0.291	IP 3269	0.243	0.279
IS 29508	0.102	0.346	ICP 16344	0.237	0.298	IP 12155	0.247	0.282
IS 2153	0.108	0.400	ICP 13999	0.242	0.293	IP 14071	0.249	0.264
IS 1128	0.109	0.378	ICP 14169	0.247	0.293	IP 7468	0.249	0.294
IS 40161	0.111	0.340	ICP 14233	0.263	0.294	IP 14418	0.250	0.278
IS 8330	0.112	0.414	ICP 15148	0.274	0.308	IP 6037	0.256	0.264
IS 21858	0.118	0.355	ICP 7621	0.274	0.292	IP 20407	0.257	0.301
IS 32252	0.118	0.370	ICP 10880	0.276	0.299	IP 13363	0.258	0.307
IS 13211	0.134	0.367	ICP 10889	0.299	0.317	IP 10705	0.265	0.276
IS 27325	0.136	0.338	ICP 12840	0.317	0.338	IP 8761	0.268	0.286
IS 31637	0.136	0.351	ICP 13545	0.410	0.384	IP 13112	0.270	0.281
IS 13215	0.141	0.395				IP 12138	0.271	0.275
Overall mean	0.090	0.387		0.203	0.302		0.227	0.271
Overall range	0.038–0.141	0.308–0.415		0.071–0.410	0.252–0.388		0.145–0.271	0.245–0.310

**Figure 2:**
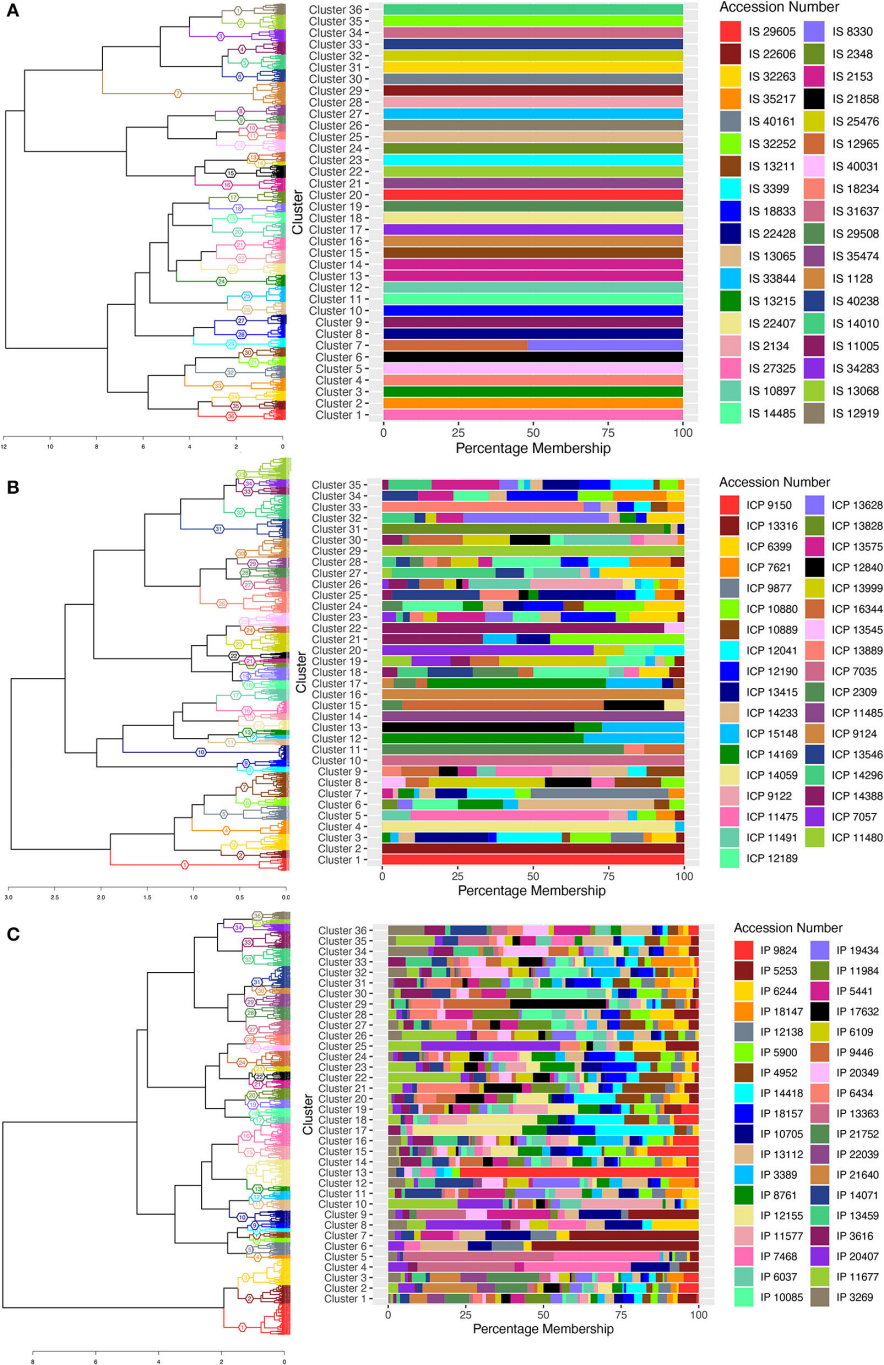
Cluster dendrogram of single plant phenotypic distances, using ward.D2 clustering algorithm, 36, 35, and 36 clusters for sorghum, pigeonpea, and pearl millet, respectively, represented with colors and cluster numbers, with percentage membership of accessions into each cluster denoted by colors in the adjacent bar graph. **(A)** the cluster dendrogram for sorghum, **(B)** the cluster dendrogram for pigeonpea, and **(C)** the cluster dendrogram for pearl millet.

Hierarchal clustering was constructed based on Gower's phenotypic distance, and the number of clusters was decided based on the number of accessions in each crop from which the data were collected. Thus, dendrogram trees were cut at 36 clusters for sorghum and pearl millet, and 35 clusters for pigeonpea, with the assumption that the individuals within accession clusters together. A cluster membership bar-plot was generated to visualize distribution or migration of individuals of different accessions to different clusters. The cluster wise stability was evaluated using the “*clusterboot*” function from the “*fpc*” package. The Jaccard coefficients between clusters of resampled data were >70 for 35 clusters in sorghum, 15 clusters in pigeonpea, and 16 clusters in pearl millet (Supplementary Table 7) and the remaining clusters showed values <70. The bootstrapped cluster dendrograms were plotted with approximately unbiased *p-*values (AU) and bootstrap probability (Supplementary Figures 1A–C) calculated using multiscale bootstrap resampling in the R-package “*pvclust*.” Bootstrap values were low in some cases of pearl millet and pigeonpea and this low bootstrap values would be a combined outcome of high variability in the data, large number of variable individuals, and the nature of clustering algorithm. Supporting the high variability and presence of valid clusters in the data, the “*pvpick*” function from the “*pvclust*” R-package yielded 66, 738, and 109 significant clusters in sorghum, pearl millet, and pigeonpea, respectively. Thus, the presence of large number of significant clusters within the studied accessions illustrates the higher variability for the observed traits and ultimately represents the higher diversity within the studied landraces. In sorghum, except cluster numbers 7, 13, and 14 all other 33 clusters have shown exclusive clustering of each accession into singleton clusters (Figure 2A). In cluster number 7, individuals of entries IS 8330 and IS 12965 were found to clustered together. The individuals of accession IS 2153 were found to be distributed in two clusters (58 individuals in cluster 14 and 139 individuals in cluster 13). In pearl millet and pigeonpea, clustering patterns showed that in most accessions, the individuals were not clustered uniquely, and found mixed with other accessions. In pigeonpea all the individuals of three accessions ICP 9150, ICP 7035, and ICP 11485 were clustered in clusters 1, 10, and 14, respectively. However, in ICP 9124, except a single individual all the other individuals were clustered in cluster 16 (Figure 2B). In pearl millet, no exclusive clusters were observed and all the 36 clusters showed mixing of individuals from different accessions (Figure 2C). However, The majority of individuals of wild accessions (IP 21640, IP 21752, and IP 22039) were distributed in 3 clusters (C-1, C-2, and C-3) showing their phenotypic similarity.

### Genotypic Diversity

**Figure 3:**
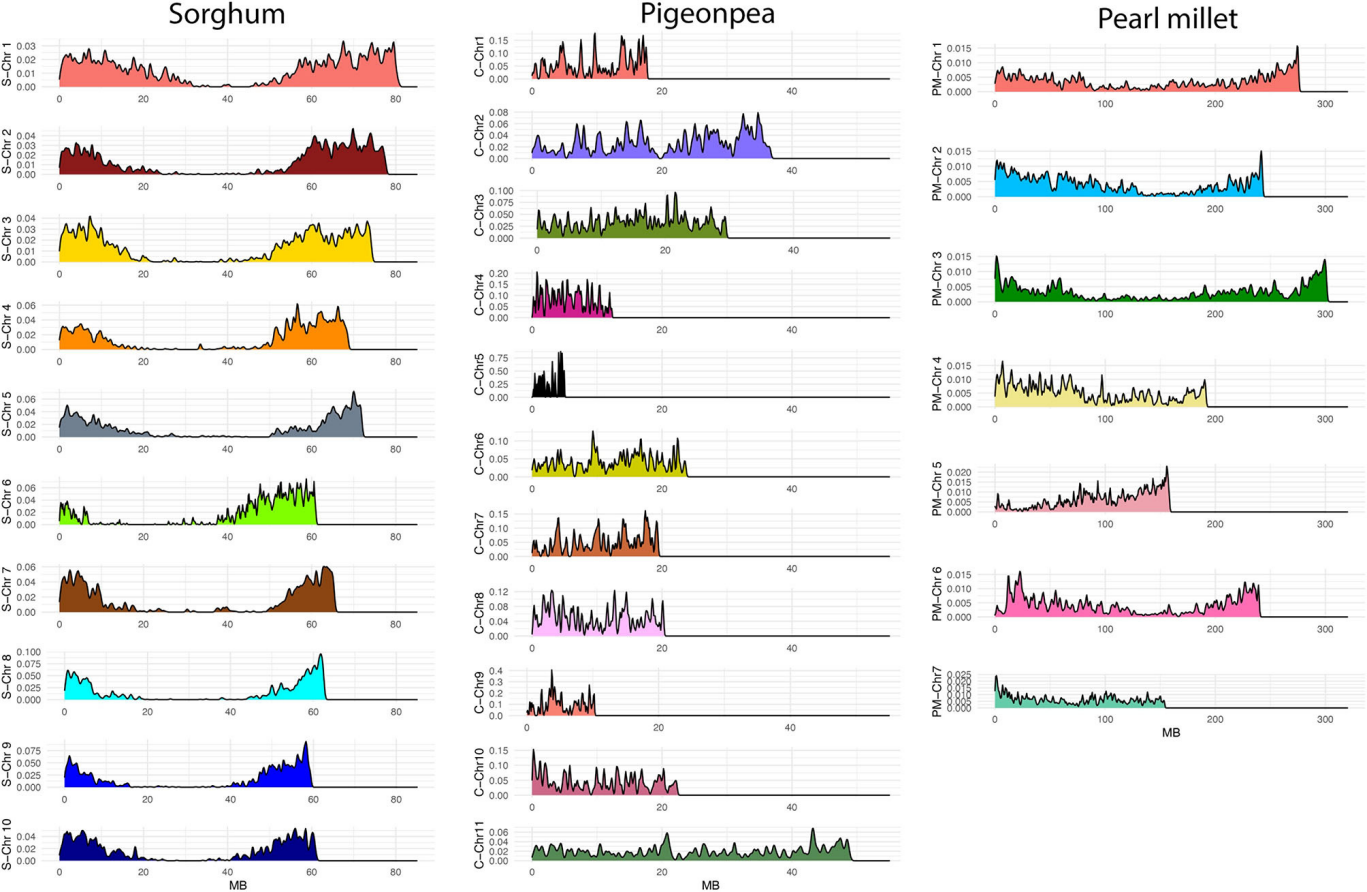
SNP densities over chromosomes of sorghum (S-Chr 1–10, the letter “S” represents sorghum), pigeonpea (C-Chr 1–11, the letter “C” represents *Cajanus*), and pearl millet (PM-Chr 1–7, the letter “PM” represents pearl millet) after filtering for quality parameters. The x-axis represents SNP positions along each chromosome (MB) and the y-axis represents SNP densities over chromosomes.

After filtering, we obtained 45,249 SNPs from a total of 76,753 SNPs in pearl millet, 19,052 SNPs from a total of 38,898 SNPs in sorghum, and 8,211 SNPs from a total of 10,096 SNPs in pigeonpea. The SNPs displayed good coverage across genome in all the three crops (Figure 3). Over the 10 chromosomes of sorghum the number of SNPs ranged from 909 to 2,988, and over the 7 chromosomes of pearl millet the number of SNPs ranged from 5,086 to 6,639, and from 121 to 755 over the 11 chromosomes of pigeonpea. The information of number of SNPs in each chromosome of sorghum, pearl millet and pigeonpea is presented in Supplementary Table 8.

### AMOVA

**Figure 4:**
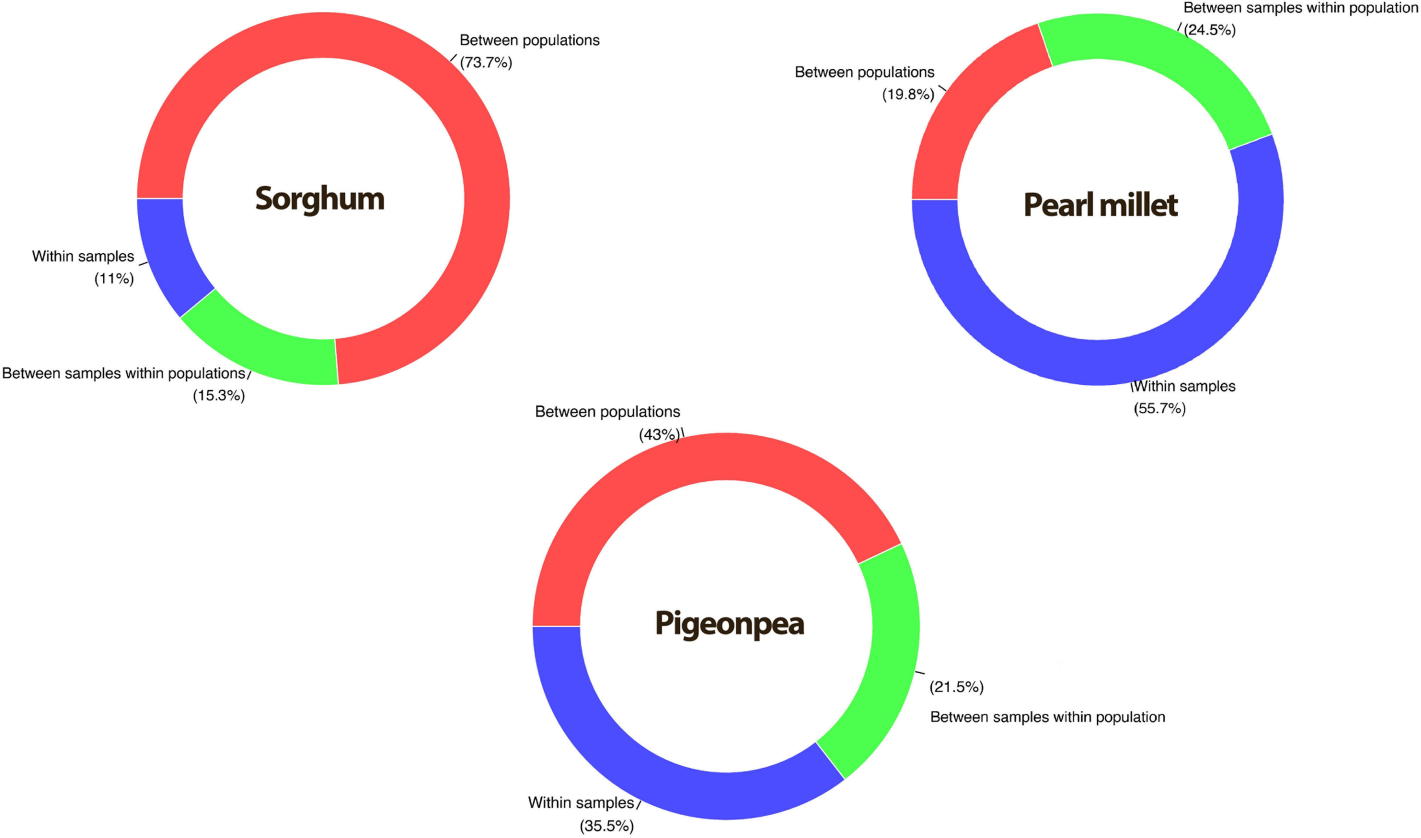
Percent contribution by different variance components in sorghum, pigeonpea, and pearl millet partitioned by AMOVA.

The analysis of molecular variance (Excoffier et al., 1992) was performed by providing predefined populations, that each accession as a separate population. The results showed that the proportion of molecular variance contributed by within accession variance depicted a low value of 26.3% in sorghum, a relatively higher value in pigeonpea (57.0%), and the highest in pearl millet (80.2%) (Table 2; Figure 4). Variance among populations was high in sorghum (73.7%), while low in pearl millet (19.8%) and intermediate in pigeonpea (43%).

**Table 2:** AMOVA on DArTSeq- SNP data of sorghum, pigeonpea, and pearl millet assuming each accession as a single population.

**Variance components**	**Df**	**Sum Sq**	**Mean Sq**	**Variance %**	**Sigma**	**Phi**	***P*-value**
Sorghum							
Between populations	35	2,508,925	71683.5	73.7	2336.9	0.8895	0.01
Between samples within populations	506	665,752	1315.7	15.3	482.8	0.5797	0.01
Within samples	542	189,717	350.0	11.0	350.0	0.7373	0.01
Total	1083	3,364,396	3106.5	100	3169.8		
Pigeonpea							
Between populations	35	368,370	10524.8	43.0	331.2	0.6449	0.01
Between samples within populations	503	304,494	605.3	21.5	165.8	0.3773	0.01
Within samples	539	147,500	273.6	35.5	273.6	0.4297	0.01
Total	1077	820,366	761.7	100	770.7		
Pearl millet							
Between populations	35	2,294,552	65558.6	19.8	1061	0.4427	0.01
Between samples within populations	981	5,507,738	5614.4	24.5	1313.1	0.3052	0.01
Within samples	1017	3,038,922	2988.1	55.7	2988.1	0.1978	0.01
Total	2033	10,841,212	5332.6	100	5362.3		

### Genotypic Diversity: Within and Between Accessions

Modified Rogers Distance (MRD) (Wright, 1978; Goodman and Stuber, 1983) between pairs of individuals were estimated. Pairwise MRD within each accession was averaged, thus the overall mean genetic distance within each accession varied from 0.031 (IS 33844) to 0.342 (IS 18833), 0.181 (IP 9824) to 0.300 (IP 22039), and 0.040 (ICP 9150) to 0.393 (ICP 10889) in sorghum, pearl millet, and pigeonpea, respectively (Table 3). Three of the five wild accessions studied in sorghum namely IS 18833 (0.342), IS 14485 (0.329), and IS 10897 (0.316), showed higher within accession distance values relative to the studied landraces and the other two wild accessions, IS 11005 (0.119) and IS 22428 (0.127), showed midrange values. However, all the studied wild accessions of pearl millet, IP 21752 (0.273), IP 21640 (0.279), and IP 22039 (0.300), showed higher within accession distance values relative to the mean distances of the pearl millet landraces studied. Averaging the MRD among accessions were found to be higher in comparison to within accession distances. Comparing the three crops, higher scale of between accession distance values were found in sorghum (0.360–0.435), followed by pigeonpea (0.237–0.422) and pearl millet (0.276–0.324).

**Table 3:** Mean genotypic within and between accession distances, observed heterozygosity within accessions (heWs) and estimated seed sample sizes using the least DArTSeq–SNP allelic frequency in all the accessions of sorghum, pigeonpea, and pearl millet.

**Sorghum**					**Pigeonpea**					**Pearl millet**				
**Accession number**	**Within accession distance**	**Distance from other accessions**	**Heterozygosity**	**No. of seeds**	**Accession Number**	**Within accession distance**	**Distance from other accessions**	**Heterozygosity**	**No. of seeds**	**Accession number**	**Within accession distance**	**Distance from other accessions**	**Heterozygosity**	**No. of seeds**
IS 10897	0.316	0.389	0.111	89	ICP 10880	0.348	0.334	0.168	89	IP 10085	0.255	0.292	0.092	173
IS 11005	0.119	0.405	0.051	101	ICP 10889	0.393	0.422	0.187	77	IP 10471	0.254	0.285	0.088	173
IS 1128	0.130	0.393	0.037	89	ICP 11475	0.201	0.283	0.058	89	IP 10705	0.265	0.286	0.104	161
IS 12919	0.038	0.381	0.032	89	ICP 11480	0.196	0.276	0.051	89	IP 11577	0.256	0.288	0.092	167
IS 12965	0.035	0.384	0.032	89	ICP 11485	0.066	0.283	0.017	89	IP 11677	0.267	0.288	0.106	161
IS 13065	0.094	0.365	0.033	89	ICP 11491	0.197	0.286	0.052	89	IP 11984	0.241	0.277	0.092	167
IS 13068	0.037	0.367	0.031	89	ICP 12041	0.169	0.242	0.043	89	IP 12138	0.270	0.285	0.103	167
IS 13211	0.145	0.400	0.047	89	ICP 12189	0.230	0.254	0.071	89	IP 12155	0.253	0.280	0.098	161
IS 13215	0.244	0.387	0.084	77	ICP 12190	0.186	0.244	0.053	89	IP 13112	0.225	0.280	0.085	179
IS 14010	0.216	0.367	0.076	83	ICP 12840	0.232	0.254	0.072	89	IP 13363	0.202	0.283	0.064	161
IS 14485	0.329	0.396	0.120	89	ICP 13316	0.228	0.272	0.123	89	IP 13459	0.225	0.285	0.085	161
IS 18234	0.034	0.379	0.024	89	ICP 13415	0.096	0.244	0.016	89	IP 14418	0.243	0.276	0.100	185
IS 18833	0.342	0.435	0.159	89	ICP 13545	0.145	0.246	0.034	89	IP 17632	0.218	0.276	0.085	167
IS 2134	0.240	0.373	0.090	89	ICP 13546	0.181	0.244	0.054	89	IP 18147	0.231	0.286	0.083	155
IS 2153	0.113	0.379	0.048	89	ICP 13575	0.185	0.245	0.053	89	IP 18157	0.251	0.291	0.088	161
IS 21858	0.104	0.376	0.044	89	ICP 13628	0.153	0.256	0.033	89	IP 19434	0.237	0.277	0.088	167
IS 22407	0.165	0.378	0.070	89	ICP 13828	0.040	0.245	0.009	89	IP 20349	0.246	0.284	0.101	197
IS 22428	0.127	0.387	0.026	89	ICP 13889	0.105	0.247	0.025	89	IP 20407	0.246	0.281	0.095	161
IS 22606	0.131	0.380	0.064	77	ICP 13999	0.210	0.253	0.058	89	IP 21640	0.279	0.310	0.134	161
IS 2348	0.085	0.390	0.043	89	ICP 14059	0.085	0.250	0.014	83	IP 21752	0.273	0.311	0.118	167
IS 25476	0.114	0.395	0.034	89	ICP 14169	0.163	0.246	0.051	89	IP 22039	0.300	0.324	0.137	161
IS 27325	0.310	0.381	0.102	47	ICP 14233	0.194	0.246	0.059	89	IP 3269	0.230	0.282	0.090	161
IS 29508	0.128	0.360	0.042	89	ICP 14296	0.166	0.246	0.040	89	IP 3389	0.249	0.282	0.098	179
IS 29605	0.099	0.361	0.036	89	ICP 14388	0.132	0.255	0.039	89	IP 3616	0.259	0.280	0.106	185
IS 31637	0.036	0.400	0.019	89	ICP 15122	0.050	0.237	0.009	89	IP 4952	0.264	0.281	0.108	173
IS 32252	0.245	0.396	0.096	89	ICP 15148	0.199	0.243	0.062	89	IP 5253	0.238	0.286	0.081	155
IS 32263	0.035	0.395	0.029	89	ICP 16344	0.204	0.268	0.063	89	IP 5441	0.248	0.280	0.101	167
IS 33844	0.031	0.381	0.024	89	ICP 2309	0.202	0.274	0.064	89	IP 5900	0.254	0.288	0.105	155
IS 3399	0.038	0.362	0.030	89	ICP 6399	0.220	0.271	0.067	89	IP 6037	0.242	0.281	0.092	173
IS 34283	0.048	0.377	0.034	89	ICP 7035	0.043	0.285	0.011	89	IP 6109	0.246	0.280	0.103	203
IS 35217	0.130	0.373	0.040	89	ICP 7057	0.143	0.265	0.043	89	IP 6244	0.262	0.286	0.102	167
IS 35474	0.109	0.364	0.040	89	ICP 7621	0.243	0.265	0.090	89	IP 6434	0.246	0.282	0.096	167
IS 40031	0.040	0.389	0.038	89	ICP 9122	0.187	0.280	0.054	89	IP 7468	0.217	0.276	0.088	167
IS 40161	0.105	0.384	0.033	89	ICP 9124	0.053	0.243	0.010	89	IP 8761	0.237	0.279	0.088	155
IS 40238	0.204	0.372	0.065	89	ICP 9150	0.040	0.241	0.007	89	IP 9446	0.226	0.278	0.084	179
IS 8330	0.129	0.382	0.056	89	ICP 9877	0.148	0.251	0.031	89	IP 9824	0.181	0.288	0.051	155
Overall mean	0.135	0.383	0.054	87		0.168	0.264	0.053	88		0.245	0.285	0.095	168
Overall range	0.031–0.342	0.360–0.435	0.019–0.159	47–101		0.04–0.393	0.237–0.422	0.007–0.187	77–89		0.181–0.3	0.276–0.324	0.051–0.137	155–203

Heterozygosity among accessions in sorghum varied from 0.019 in IS 31637 to 0.159 in IS 18833. Among five wild accessions studied in sorghum, three accessions had higher heterozygosity (0.111 in IS 10897, 0.120 in IS 14485, and 0.159 in IS 18833) in comparison to all the landraces, while the other two wild accessions had low heterozygosity (0.026 in IS 22428 and 0.051 in IS 11005). In pearl millet, IP 9824 (0.051) and IP 22039 (0.137) estimated the lowest and highest heterozygosity, respectively. As in sorghum, the wild accessions of pearl millet *viz.*, IP 21640 (0.134), IP 21752 (0.118), and IP 22309 (0.137) depicted maximal heterozygosity estimates in comparison to the landraces. In pigeonpea, ICP 9150 (0.007) had the minimum heterozygosity while ICP 10889 (0.187) had the highest heterozygosity (Table 3).

**Figure 5:**
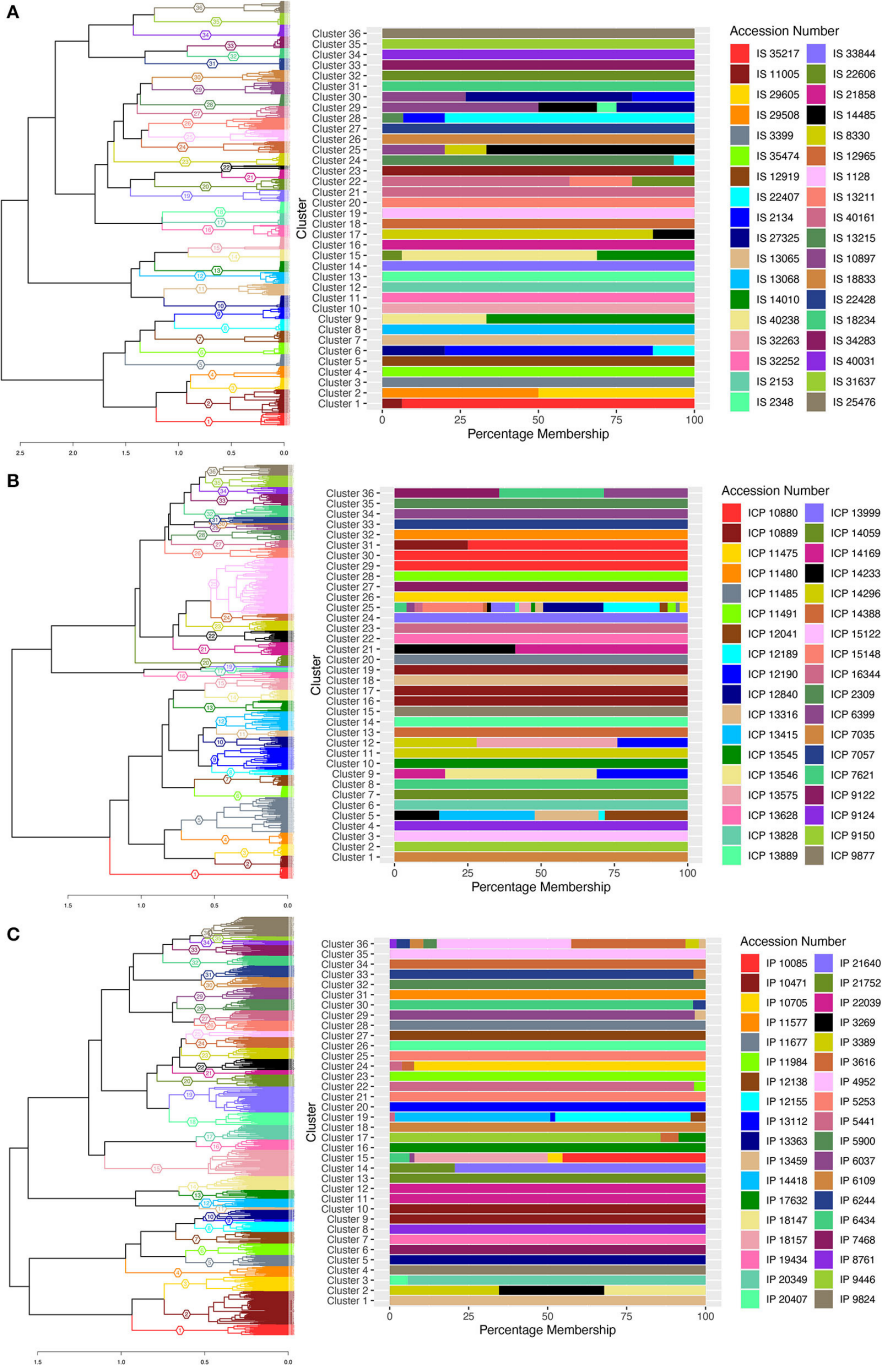
Cluster dendrogram of single plant genotypic distances estimated from DArTSeq- SNP data, using ward.D2 clustering algorithm, 36 clusters for sorghum, pigeonpea, and pearl millet, represented with colors and cluster numbers, with percentage membership of accessions into each cluster denoted by colors in the adjacent bar graph. **(A)** the cluster dendrogram of sorghum, **(B)** the cluster dendrogram of pigeonpea, and **(C)** the cluster dendrogram of pearl millet.

Clustering based on MRD grouped the individuals of all the accessions into different clusters. The cluster wise stability was assessed using the “*clusterboot*” function from R-package “*fpc*” with 100 bootstraps. About 33 clusters in sorghum, 24 clusters in pigeonpea and 19 clusters in pearl millet showed Jaccard coefficient values >70 and all the other clusters showed values <70. The Jaccard coefficient values of all the clusters were presented in Supplementary Table 9. Bootstrapped dendrograms (Supplementary Figures 2A–C) with 100 bootstraps were plotted using the “*aboot*” function provided in the R-package “*poppr*.” The dendrogram tree was cut at 36 clusters considering number of accessions in the respective crops, with an assumption that the individuals of each accession should aggregate into singleton cluster. Also a cluster membership bar-plot was used to visualize this cluster partition and migration of plants to different clusters. In sorghum, 19 accessions, *viz.*, IS 1128, IS 12919, IS 12965, IS 13065, IS 18234, IS 18833, IS 2153, IS 21858, IS 22428, IS 25476, IS 31637, IS 32252, IS 32263, IS 33844, IS 3399, IS 34283, IS 13068, IS 35474, and IS 40031 were found to be uniform, by clustering of all the individual of an accessions into separate singleton clusters (Figure 5A), while the landraces IS 29508 and IS 29605 were found to be grouped in a single cluster. All other accessions of sorghum were found to have mixtures. In pigeonpea, ICP 2309, ICP 9124, ICP 7057, ICP 9877, ICP 11480, ICP 14059, ICP 13628, ICP 7035, ICP 9150, ICP 13828, and ICP 15122 showed perfect singleton clustering (Figure 5B), while other accessions showed overlapping of individuals of different accessions which may be explained due to the heterogeneity achieved in evolutionary gene-flow or the presence of admixtures in the respective accessions. In pearl millet, a completely distinctive and complex distribution of accessions into clusters has been noticed. The accessions IP 9824, IP 7468, IP 11577, IP 11677, IP 13363, and IP 19434 showed perfect singleton clustering (Figure 5C) while all other accessions were not clustered uniquely to singleton clusters, indicating heterogeneity within landraces and sharing of alleles between accessions. The wild accessions of pearl millet showed an interesting pattern of clustering that the individuals of the accession IP 22039 was shared between cluster numbers 11 and 12 showing the presence of a variable set of alleles or two subpopulations, and also there can be seen some individuals of the accession IP 21752 clustered with the individuals of the accession IP 21640 in cluster number 14 depicting some similar alleles between these two accessions.

### Shannon Diversity

The Shannon diversity was estimated for all 36 accessions in sorghum, pigeonpea, and pearl millet (Table 4). The values ranged from 0.113 to 2.363 with an overall mean of 0.275 in sorghum, 0.121–1.739 with an overall mean of 0.498 in pigeonpea and 1.128–2.715 with an overall mean of 1.856 in pearl millet. In sorghum, the accession IS 22606 (0.113) had the lowest Shannon diversity followed by the accessions IS 35474 (0.114) and IS 35217 (0.116), and the accession IS 18833 (2.363) had the highest Shannon diversity followed by accessions IS 32252 (1.138) and IS 14485 (0.583). In pigeonpea the accession ICP 9150 (0.121) had the lowest value of Shannon diversity followed by the accessions ICP 15122 (0.148) and ICP 13415 (0.156), and the accession ICP 13316 (1.739) had the highest Shannon diversity followed by the accessions ICP 10880 (1.542) and ICP 10889 (1.404). In pearl millet the accessions IP 9824 (1.128), IP 10471 (1.270), and IP 18157 (1.335) had lower values of Shannon diversity and the accessions IP 21640 (2.715), IP 6109 (2.677), and IP 20349 (2.489) had higher values. It should be noted that, in both sorghum and pearl millet, all the wild accessions had high values of Shannon diversity. Three out of five wild accessions in sorghum IS 18833 (2.363), IS 14485 (0.583), and IS 10897 (0.495) had higher values relative other accessions of sorghum, while the remaining two wild accessions IS 22428 (0.192) and IS 11005 (0.198) found to have intermediate values. Also, the wild accessions of pearl millet had relatively higher values of Shannon diversity IP 21640 (2.715), IP 22039 (2.382), and IP 21752 (2.359) in comparison to all other accessions of pearl millet.

**Table 4:** Shannon diversity (H′) estimates within each accession of sorghum, pigeonpea, and pearl millet estimated from the DArTSeq–SNPs.

**Sorghum**		**Pigeonpea**		**Pearl millet**	
**Accession number**	**H^**′**^**	**Accession number**	**H^**′**^**	**Accession number**	**H^**′**^**
IS 22606	0.113	ICP 9150	0.121	IP 9824	1.128
IS 35474	0.114	ICP 15122	0.148	IP 10471	1.270
IS 35217	0.116	ICP 13415	0.156	IP 18157	1.335
IS 13068	0.117	ICP 13828	0.161	IP 13363	1.357
IS 14010	0.117	ICP 9124	0.167	IP 11577	1.408
IS 18234	0.119	ICP 14059	0.168	IP 5253	1.430
IS 3399	0.120	ICP 7035	0.212	IP 12138	1.458
IS 33844	0.120	ICP 9877	0.239	IP 10085	1.518
IS 12965	0.120	ICP 13628	0.261	IP 8761	1.531
IS 12919	0.123	ICP 14296	0.282	IP 18147	1.664
IS 34283	0.128	ICP 11485	0.312	IP 13459	1.685
IS 31637	0.129	ICP 11491	0.313	IP 10705	1.706
IS 13065	0.131	ICP 13889	0.318	IP 12155	1.706
IS 40238	0.132	ICP 13545	0.321	IP 11677	1.709
IS 22407	0.132	ICP 9122	0.342	IP 6037	1.710
IS 32263	0.134	ICP 13999	0.397	IP 6244	1.758
IS 25476	0.135	ICP 14388	0.398	IP 20407	1.797
IS 40031	0.136	ICP 12041	0.404	IP 19434	1.802
IS 40161	0.138	ICP 11480	0.419	IP 11984	1.809
IS 1128	0.147	ICP 13575	0.449	IP 9446	1.925
IS 2348	0.155	ICP 6399	0.449	IP 5900	1.952
IS 29605	0.159	ICP 14233	0.473	IP 6434	1.972
IS 13211	0.167	ICP 12190	0.532	IP 7468	1.975
IS 29508	0.179	ICP 11475	0.541	IP 17632	1.978
IS 8330	0.185	ICP 12840	0.544	IP 5441	2.019
IS 2134	0.189	ICP 15148	0.561	IP 3389	2.026
IS 22428	0.192	ICP 12189	0.575	IP 3269	2.059
IS 11005	0.198	ICP 13546	0.587	IP 4952	2.076
IS 2153	0.234	ICP 16344	0.623	IP 3616	2.133
IS 27325	0.288	ICP 7057	0.626	IP 13112	2.256
IS 21858	0.390	ICP 2309	0.667	IP 21752	2.359
IS 13215	0.494	ICP 14169	0.679	IP 14418	2.379
IS 10897	0.495	ICP 7621	0.798	IP 22039	2.382
IS 14485	0.583	ICP 10889	1.404	IP 20349	2.498
IS 32252	1.138	ICP 10880	1.542	IP 6109	2.677
IS 18833	2.363	ICP 13316	1.739	IP 21640	2.715
Overall mean	0.275		0.498		1.865
Overall range	0.113–2.363		0.121–1.739		1.128–2.715

### Relationship Between Phenotypic and Genotypic Distances

Mantel's correlation between phenotypic and genotypic distance matrices showed highly significant positive correlation (*r* = 0.45, *P* ≤ 0.01) for sorghum, pearl millet (*r* = 0.13, *p* ≤ 0.01), and pigeonpea (*r* = 0.19, *P* ≤ 0.01), thus depicting the effectiveness of complimentary use of molecular and phenotypic tools as a better approach for the assessment of the genetic diversity.

### Population Structure Using DAPC

Detecting the number of clusters using the *find.cluster* function hasn't shown any significant elbow of reduction in BIC values (Supplementary Figure 3), instead, a gradual reduction in the BIC values was seen on increasing number of clusters. So that, DAPC was carried out using 36 clusters representing the 36 accessions in sorghum, pearl millet and pigeonpea. The *optm.a.score* function detected an optimal first 45 PCs for pearl millet and first 7 PCs for both sorghum and pigeonpea. Based on the posterior membership probabilities the population membership graph showing the population structure was created. In sorghum (Supplementary Figure 4A), 17 accessions (IS 1128, IS 12965, IS 18234, IS 18833, IS 2153, IS 21858, IS 25476, IS 31637, IS 32252, IS 32263, IS 33844, IS 3399, IS 34283, IS 35217, IS 35474, IS 40031, and IS 40161) were clustered exclusively into separate populations, whereas both the accessions IS 29508 and IS 29605 were clustered into a single population and all the other accessions are seen to have mixtures at different levels. In the population structure of pigeonpea (Supplementary Figure 4B), seven accessions (ICP 13828, ICP 14059, ICP 7035, ICP 11485, ICP 14169, ICP 15122, and ICP 9150) were found to be pure, and in pearl millet (Supplementary Figure 4C), seven accessions (IP 6434, IP 7468, IP 9824, IP 18157, IP 13363, and IP 3389) were seen to be clustered perfectly without any posterior probability for assessment to other populations while all the other accessions in both pearl millet and pigeonpea have a considerable amount of mixtures depicting the heterogeneity in the respective accessions.

For all the 36 populations, the quality of the attribution of accessions into populations were investigated by estimating the *a-scores* (Supplementary Table 10). An *a-scores* of 1 represents an accurate allocation of the plants into groups. Twenty-seven clusters (1, 3, 4, 5, 6, 7, 8, 10, 11, 13, 14, 15, 17, 18, 19, 21, 22, 23, 24, 27, 28, 29, 30, 33, 34, 35, and 36) in sorghum, 3 clusters in pearl millet (4, 24, and 30) and 17 clusters in pigeonpea (1, 2, 10, 11, 12, 13, 14, 15, 16, 17, 21, 22, 24, 25, 29, 32, and 33) showed a greater reliability (a-score = 0.81–0.99). The clusters (2, 9, 12, 16, 26, and 31) in sorghum, the clusters (2, 5, 6, 8, 10, 12, 13, 15, 16, 17, 18, 19, 20, 21, 25, 26, 27, 28, and 29) in pearl millet and the clusters (4, 5, 6, 7, 9, 18, 19, 20, 23, 26, 30, 31, 34, and 36) in pigeonpea showed an average reliability (a-score = 0.65–0.80) in the attribution, whereas 3 clusters (20, 25, and 32) from sorghum, 14 clusters (1, 3, 7, 9, 11, 14, 22, 23, 31, 32, 33, 34, 35, and 36) from pearl millet and 5 clusters (3, 8, 27, 28, and 35) from pigeonpea were found to have a low reliability (a-score = <0.65) in the attribution to the DAPC detected populations.

### Estimation of Seed Sample Size

**Figure 6:**
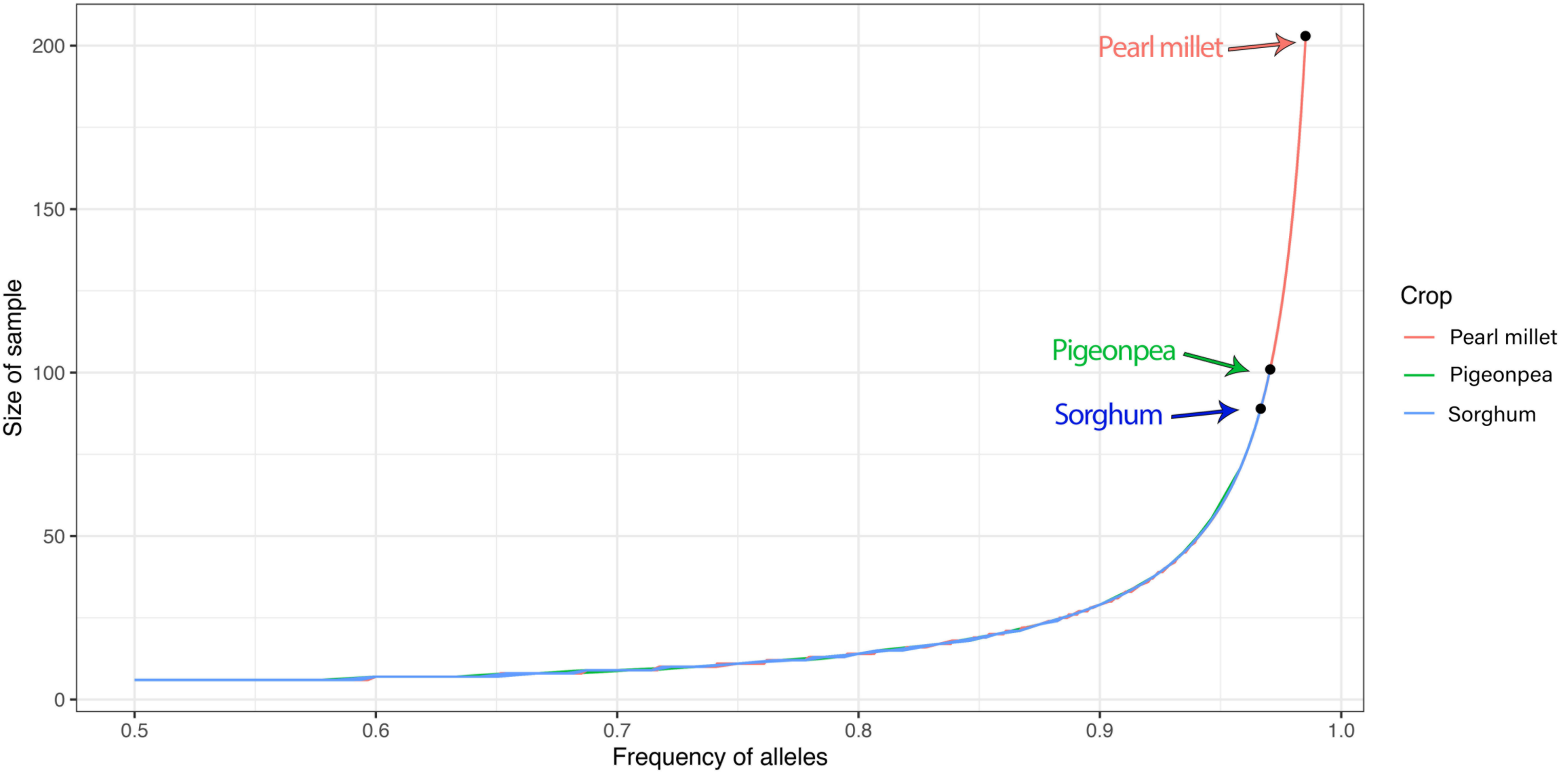
Plot of seed sample sizes needed for regeneration vs. allelic frequencies to preserve 95% of alleles showing the exponential increase in the size of sample required with the decrease in the alternate allele frequency. The arrows represent the highest values of seed sample size estimated in sorghum, pigeonpea, and pearl millet.

Seed sample sizes required for regeneration to capture 95% of the alleles with an expected probability of 95%, was estimated based on the allelic frequencies of the DArTSeq–SNPs, for each accession using the model proposed by Crossa (1989). The results of the sample sizes required are given in Table 3. Seed sample sizes for sorghum ranged from 47 to 101, 155 to 203 for pearl millet, and 77 to 89 for pigeonpea. The seed sample size increments exponentially after the alternate allele frequency attains a value below 0.1 (Figure 6) depicting the need for an exponentially larger sample size for conserving the alleles with frequencies below 0.1. The number of rare allelic variants or markers (frequency less than or equal to 5% within accessions) preserved in the recommended sample size for each accession of the three crops (Supplementary Table 11) ranged from 345 to 3,075 in sorghum, 231 to 878 in pigeonpea, and 3,444 to 6,726 in pearl millet.

## Discussion

Sorghum, pearl millet, and pigeonpea are the important food crops, providing food and income to a large population thriving in the arid and semi-arid tropics. However, in this era of modern agriculture, landraces of these crops are becoming prone to genetic erosion, (Hammer et al., 1996; Shewayrga et al., 2008; Pattanashetti et al., 2016). Most landraces, that were permanently extinct from the farmers' field over the course of agricultural development, are only available in genebanks' collections. As each landrace possess a unique genetic fingerprint of ages of acclimatization to diverse environmental conditions, they are considered as an indispensable source of genetic variations by plant breeders and can address a potential scope in the development of improved varieties with higher productivity, nutrients, and climate resilience, etc. (Dwivedi et al., 2016). Thus, conserving landraces with their inherent genetic variability is crucial for ensuring food security in the near future and also for sustainable agriculture. ICRISAT genebank conserves about 42,000 accessions of sorghum, 24,000 accessions of pearl millet, and over 13,000 accessions of pigeonpea, wherein about 86% of sorghum and pearl millet collections and over 60% of pigeonpea collections are landraces. The main focus of genebank curator is to maintain the genetic integrity and diversity within accessions while regeneration. Hence, this study assessed the diversity within landrace accessions by phenotyping and genotyping a large number of plants within each accession and estimated the seed sample size required in order to conserve the inherent diversity.

Enormous variability was observed within and among landraces of sorghum, pigeonpea, and pearl millet. Molecular variance within accessions was observed to be low in sorghum (26.3%), highest in pearl millet (80.2%), while pigeonpea showing an intermediate within accession variance of 57.0%. Our results are in correspondence with previous works, by various authors (Tostain et al., 1987; Tostain and Marchais, 1989; Busso et al., 2000; Bashir et al., 2015) on pearl millet landraces, reported a high intra-population variation of 70–90% and higher observed heterozygosity of 0.77–0.82. However, Bhattacharjee et al. (2002) reported a low 30.89% within accession variability using RFLP markers in pearl millet, also the author addressed this low variability as a contradiction for a cross-pollinated crop like pearl millet and discussed various instances that would have caused this lower variability. In sorghum, Adugna (2014) reported a 54.44% molecular variance due to diversity within landrace populations that were conserved on farms in Ethiopia. No studies investigating landraces diversity within accessions were reported in sorghum and pigeonpea, while few studies are on landraces conserved on-farm that are continually evolving through outcrossing and selections (Djè et al., 1999; D'Andrea and Casey, 2002; Songok et al., 2010; Adugna, 2014; Bashir et al., 2015).

**Figure 7:**
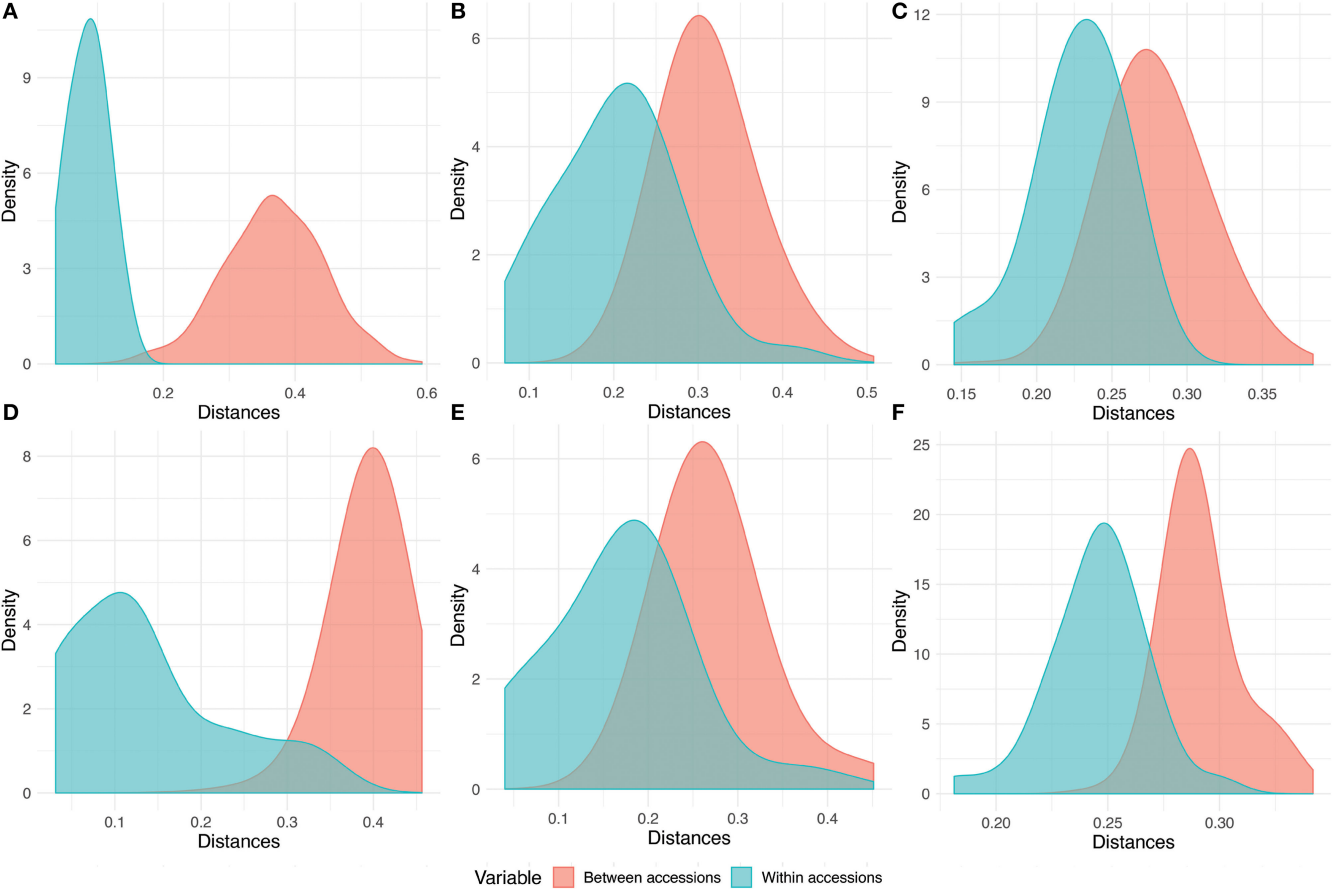
Comparison graph of densities of within and between accession distances. **(A–C)** denotes the phenotypic distance distribution for sorghum, pigeonpea, and pearl millet, respectively, and **(D–F)** denotes the genotypic within and between accession distance distribution estimated from DArTSeq-SNP data of sorghum, pigeonpea, and pearl millet, respectively.

The phenotypic and genotypic within accession distances were scaled toward the higher values in pearl millet, so that blurring the differentiation of within and between accessions diversity. The density distribution of within and between accession distances in pearl millet showed this scenario clearly, exhibiting the merging of densities (Figures 7C,F) of within and between accession distances in both phenotypic and genotypic evaluation. Pigeonpea being often cross-pollinated also depicted a pattern of overlapping within and among accession distances in both phenotypic and genotypic evaluation (Figures 7B,E). Whereas, sorghum showed a clear separation of distances within accessions from distances between accessions in both phenotypic and genotypic assessment, depicting the higher uniformity and homogeneity within the accessions (Figures 7A,D). The higher values and merging of between and within accession distances in pearl millet and pigeonpea shows the high phenotypic and genotypic heterogeneity within accessions, and also the clear separation of densities of within and between accession distances in sorghum clearly explains the higher uniformity within the accessions of sorghum.

Population structure analyses indicated that most of the accessions in sorghum were uniform enough to cluster individuals of single accession together as a singleton clusters. In sorghum, accessions IS 29508 and IS 29605 were clustered into a single population in both DAPC and ward.D2 clustering with genotypic distances, indicating the presence of high similarity and common alleles in these two accessions. The accession IS 33844 showed high uniformity with a low within accession diversity (0.031), and a selection from this landrace has been released as a variety in India as ‘Parbhani moti’ (Upadhyaya and Vetriventhan, 2018). Pearl millet and pigeonpea showed a higher heterogeneity within accessions, while most of the accessions showed mixed populations. In pigeonpea and pearl millet only some accessions showed singleton clustering. High population mixtures in these crops correspond to their pollination behavior, and sharing of alleles between populations. Landraces generally differ between populations, based on the intensity of selection imposed by farmers, their pollination mechanisms, the level of gene-flow within and between population, and level of exchange of seed materials between farmers. Previously several authors reported pollen flow between populations and the mixing of landrace populations in sorghum and pigeonpea (Songok et al., 2010; Kassa et al., 2012; Adugna, 2014; Westengen et al., 2014). Harlan (1965) reported the gene-flow from weeds to landraces and several other authors (Ellstrand et al., 1999; Jarvis and Hodgkin, 1999; Messeguer, 2003; Gompert and Buerkle, 2016) reported the transfer of genes into landraces from various sources in both self and outcrossing species. Also, some studies reported the mixing of the population by a considerable exchange of seeds within cultures or regions (Louette, 1997). The level of heterogeneity and diversity in landraces are crop-specific and associated with their mode of fertilization (Villa et al., 2005) and also several authors (Hammer et al., 1996; Zeven, 1998) stated the influence of mode of pollination in various population genetics factors over the course of evolution of landraces. Hence, complying to the effect of mode of reproduction on diversity, a higher degree of outcrossing (about 85%) in pearl millet (Burton, 1983) could impose a higher diversity in pearl millet, in comparison to lower outcrossing crops such as sorghum (about 18%) (Barnaud et al., 2008) and an intermediate outcrossing crops (about 30%) like pigeonpea (Saxena et al., 1990), and this varies with species.

Most of the accessions that showed relatively higher within genetic distances in sorghum and pearl millet were wild accessions. Thus, using wild accessions in this study helped us in the comparative assessment with landraces and also aided in the better understanding of the effect of domestication and different evolutionary forces that shaped the landraces. Historically farmers conserving landraces on-farm and multiplied desirable phenotypes, which survived both natural and artificial selection. The effect of this farmers' selection led to local adaptations and variations within the landrace populations (Zeven, 1998). Teshome et al. (2016) studied the maintenance of landrace diversity in sorghum by farmers belonging to different regions in Ethiopia and reported a narrow preference to specific economic traits and selection by farmers. Thus, the wild accessions in this case lack of farmers' selections and its obligatory to be highly diverse as these are evolving under natural selection.

**Figure 8:**
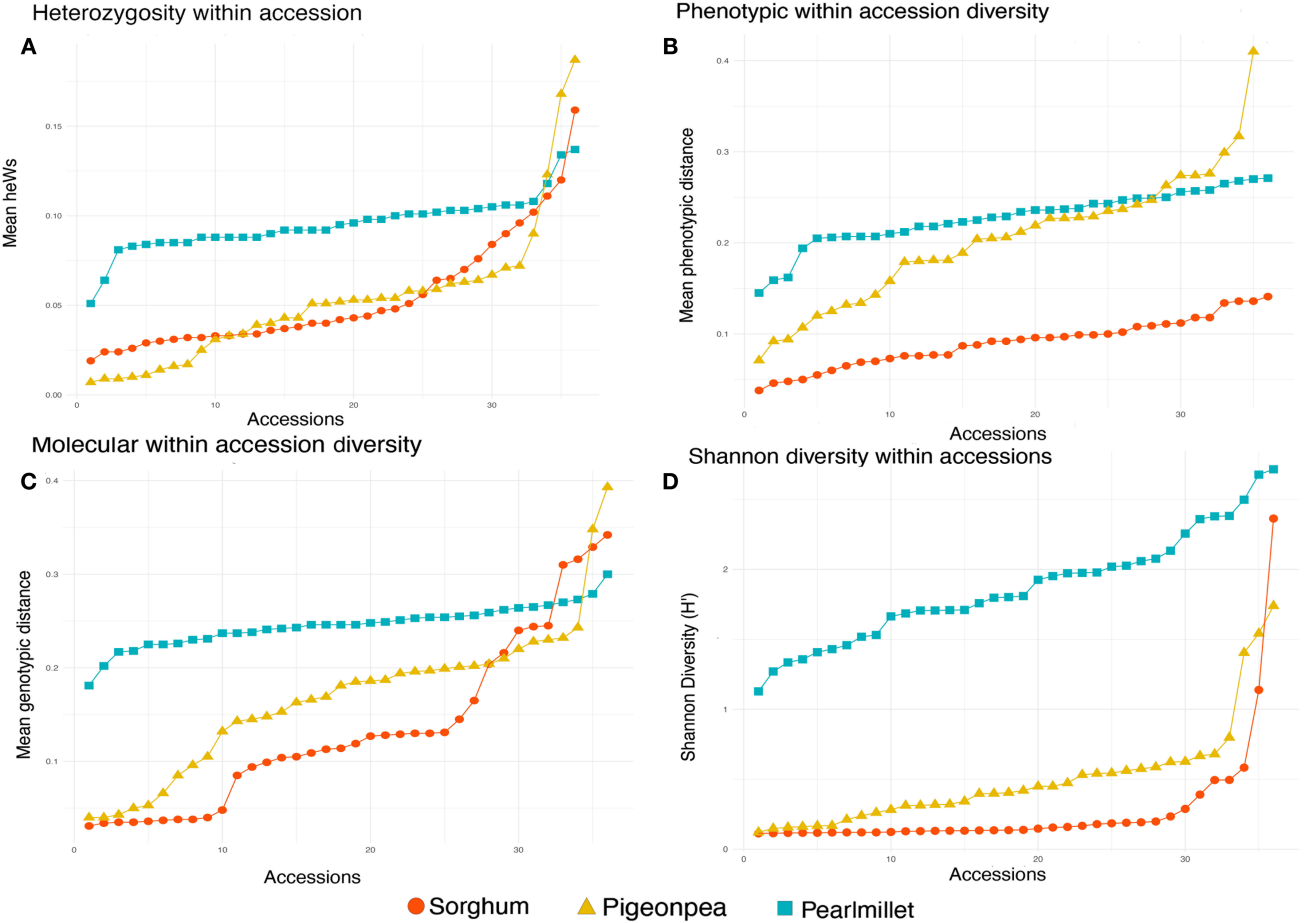
**(A)** Average heterozygosity within accessions for each 36 accessions of sorghum, pigeonpea, and pearl millet estimated from DArTSeq SNP data, **(B)** Average phenotypic within accessions distances for 36 accessions of sorghum and pearl millet and 35 accessions of pigeonpea, **(C)** Average genotypic within accession distances for each 36 accessions of sorghum, pigeonpea, and pearl millet estimated from DArTSeq SNP data, **(D)** Shannon diversity within accessions for each 36 accessions of sorghum, pigeonpea, and pearl millet estimated from DArTSeq SNP data.

Comparing diversity of the three crops in our study, heterozygosity (Figure 8A), phenotypic (Figure 8B), and genotypic (Figure 8C) within accession diversity of sorghum were notably low for most of its accessions, intermediate for most of the accessions of pigeonpea and followed a more stable trend around the maximal values for pearl millet. Similar to the molecular within accession distances, Shannon diversity revealed diversity estimates, scaled over the higher values for pearl millet, followed by an intermediate in pigeonpea and lower estimates for sorghum (Figure 8D). However, in sorghum and pigeonpea both highly diverse and highly uniform accessions with maximal and minimal estimates of genotypic distances and Shannon diversity were observed. The higher diversity estimates indicate the presence of higher variability within accessions. In case of pigeonpea most of the accessions were found to have molecular within accession distances <0.250 except two accessions *viz.*, ICP 10880 (0.348) and ICP 10889 (0.393). On further investigation into the individual plant within accession distances of these accessions, it appeared that, some individuals within these accessions were diverse from all the other individuals of the respective accession. Such that, the accession ICP 10880 had two individuals that were highly divergent from all other individuals by a mean distance of 0.410 and 0.434. Also these individuals were found to cluster separately in hierarchal clustering. Same for the accession ICP 10889, where some individuals were highly divergent from the other. In case of sorghum, most of the accessions had a molecular within accession distances <0.250 except three wild accessions *viz.*, IS 10897 (0.316), IS 14485 (0.329), IS 18833 (0.342) and one landrace IS 27325 (0.310). In the landrace IS 27325, it can be seen that the individuals are divided into three subgroups in hierarchal clustering. Thus, higher diversity in some landraces of sorghum and pigeonpea can be due their pollination behavior, which ultimately influences the population substructure. The lower outcrossing in these crops offers the higher probability of fixation of various alleles within a fewer members or individuals, restricting the frequency/occurrence of some allele within a small group of a landrace population, thus gradually over generations, forming distinct subpopulations within groups. These varied groups of individuals are however not phenotypically variable enough to consider it as separate population, but however assimilated a genetically distinct fingerprint from various elements throughout the course of evolution. Similar cases of extreme values of low and high diversity were previously encountered by researchers. Zeven (1998) emphasized the low diversity and increased homozygosity in inbreeding accessions and also explained the influence of farmers' selection and sampling strategies for reduction of diversity in landrace populations. Adugna (2014) and Westengen et al. (2014) found both high and low within-population diversity in sorghum landraces cultivated in Ethiopia and reasoned the low within landrace diversity could be due to farmers' sampling during migration, as farmers tend to carry few heads during migration and settlements.

Based on the level of diversity within each accession of different crops, appropriate conservation and regeneration strategy should be followed to conserve the genetic integrity and diversity of landraces. ICRISAT genebank follows various pollination control and sampling strategies to maintain the genetic integrity and diversity within accessions, while regenerating different crops. Theoretically, selfing will be a good strategy to maintain the genetic integrity and diversity in self-pollinated crops and often-cross pollinated crops (out-crossing >5%), because of the low effect of inbreeding depression, and to preserve alleles within the population. In cross-pollinated species like pearl millet, sib mating is the best strategy to mimic the random mating, and for that ICRISAT genebank performs cluster bagging (bagging few panicles of different individuals of the same accession) that reduces the effect of inbreeding depression. However, in both cases, the appropriate population size needs to be ensured while regeneration for capturing the rare alleles. Small sample sizes while regenerating landraces may lead to genetic drift which results in the loss of some rare alleles. Crossa (1989) based on his results on stimulated populations, reported a practical system for maize regeneration, wherein the author discussed that the ideal system of regeneration involves equalizing the genetic contribution of parents and avoiding small population sizes and, also Crossa (1995) suggested a practical seed sample size of 130–200 in monoecious crops for retaining the rare alleles in most of the loci. FAO standards specify a sample size of 30 individuals in a completely random mating population and 60 individuals for completely selfing species to capture 95% of the alleles which have a frequency >0.05 (FAO, 2014). However, in sorghum, pigeonpea, and pearl millet, no detailed molecular studies were done previously utilizing NGS tools to determine optimum population size requirements for regeneration. Therefore, we estimated the minimum sample size to capture 95% of the SNP alleles spread throughout the whole genome with an expected probability of 95% based on the least frequent allele or the frequency of the rarest allele for each accession following Crossa (1989). From our study, seed sample sizes were found to be minimal for sorghum (47–101), and pigeonpea (77–89), and high for pearl millet (155–203). The sample size required to conserve the genetic integrity of germplasm depends largely on the frequency of the least common alleles or genotypes.

In conclusion, sorghum, pigeonpea, and pearl millet accessions showed higher within and among accession diversity, indicating that the regeneration strategies at ICRISAT genebank are appropriate to ensure the genetic integrity of each accession. Information from this study will support genebank curators in understanding within accession variability and assists in devising scientific sampling strategies (sample size) for regeneration to maintain the genetic integrity and variability. This could also help breeders in the utilization end to understand the population dynamics and subpopulation structure, to forward the material with appropriate breeding techniques.

## Data Availability Statement

The original contributions presented in the study are publicly available. This data can be found here: http://dataverse.icrisat.org/dataset.xhtml?persistentId=doi:10.21421/D2/CCSOZ8 for pigeonpea http://dataverse.icrisat.org/dataset.xhtml?persistentId=doi:10.21421/D2/WU4JFA for pearl http://dataverse.icrisat.org/dataset.xhtml?persistentId=doi:10.21421/D2/DSYLHB for sorghum.

## Author Contributions

VCRA, MV, SD, and AR contributed to conception and design of the study. VCRA, MV, VA, RS, VK, PS, and SR conducted field experiments and data collection. This work is part of VA's thesis research. SG supported student research as chairman. VA and MV performed the statistical analysis and wrote the first draft of the manuscript. VCRA reviewed and approved the first draft. All authors contributed to manuscript revision, read, and approved the submitted version.

## Conflict of Interest

The authors declare that the research was conducted in the absence of any commercial or financial relationships that could be construed as a potential conflict of interest.

## Supplementary Material

Supplementary Figure 1 Cluster dendrogram with unbiased bootstrap probability values for edges, with ward.D2 clustering for Gower's distances, for single plant phenotypic data **(A)** The cluster dendrogram of sorghum, **(B)** the cluster dendrogram of pigeonpea, and **(C)** Cluster dendrogram of pearl millet.

Supplementary Figure 2 Cluster dendrogram with bootstrap probability values for edges, with ward.D2 clustering for Modified Roger's distances, for single plant genotypic data **(A)** The cluster dendrogram of sorghum, **(B)** the cluster dendrogram of pigeonpea, and **(C)** Cluster dendrogram of pearl millet.

Supplementary Figure 3 Values of BIC vs. number of clusters with maximum of 70 clusters in DAPC analysis for **(A)** sorghum, **(B)** pigeonpea, and **(C)** pearl millet.

Supplementary Figure 4 Population structure using the posterior membership probabilities, using K = 36 in DAPC analysis: **(A)** The population structure of sorghum. **(B)** Population structure of pigeonpea and **(C)** Population structure of pearl millet.

Supplementary Table 1 List of accessions of sorghum used for phenotypic and genotypic within accession diversity evaluation.

Supplementary Table 2 List of accessions of pigeonpea used for phenotypic and genotypic within accession diversity evaluation.

Supplementary Table 3 List of accessions of pearl millet used for phenotypic and genotypic within accession diversity evaluation.

Supplementary Table 4 List of the quantitative and qualitative traits recorded in sorghum, pearl millet and pigeonpea.

Supplementary Table 5 Mean grouping by Student-Newman-Keuls Test of sorghum, pearl millet and pigeonpea for all the quantitative traits recorded.

Supplementary Table 6 Levene's test for significant differences in population variances.

Supplementary Table 7 Bootstrapping values (Jaccard coefficient) of clusters for phenotypic data.

Supplementary Table 8 Number of DArTSeq-SNPs in each chromosome of sorghum, pearl millet and pigeonpea after filtering.

Supplementary Table 9 Bootstrapping (Jaccard coefficients) values of clusters for DArTSeq SNP data.

Supplementary Table 10 Probability of attribution of all the single plants in each accession into different groups based on the discriminant analysis of principle components on DArTSeq-SNP data (K = 36).

Supplementary Table 11 Number of rare alleles preserved in the recommended sample size estimated from DArTSeq-SNP data of sorghum, pigeonpea, and pearl millet.
