# Supplementary material for: Genome-Wide DArTSeq Genotyping and Phenotypic Based Assessment of Within and Among Accessions Diversity and Effective Sample Size in the Diverse Sorghum, Pearl Millet, and Pigeonpea Landraces
Source: Front Plant Sci. 2020 Dec 14;11:587426. doi: 10.3389/fpls.2020.587426 (PMC7768014; doi:10.3389/fpls.2020.587426)
Supplement: Supplementary Figure 1 — Cluster dendrogram with unbiased bootstrap probability values for edges, with ward.D2 clustering for Gower's distances, for single plant phenotypic data (A) The cluster dendrogram of sorghum, (B) the cluster dendrogram of pigeonpea, and (C) Cluster dendrogram of pearl millet. [file Data_Sheet_1.zip › Supplemantary material_corrected/Table S5.docx]

| Acc.No | Days to flowering | groups | Acc.No | Days to maturity | groups | Acc.No | Plant height | groups | Acc.No | Leaf blade length(Cm) | groups | Acc.No | Leaf blade width (Cm) | groups | Acc.No | Panicle excersion (Cm) | groups | Acc.No | Panicle length (Cm) | groups |
| --- | --- | --- | --- | --- | --- | --- | --- | --- | --- | --- | --- | --- | --- | --- | --- | --- | --- | --- | --- | --- |
| IS 22606 | 92.07 | a | IS 22606 | 138.46 | a | IS 22606 | 309.158621 | a | IS 22606 | 78.80 | a | IS 22606 | 7.83 | a | IS 22428 | 22.38 | a | IS 22606 | 46.02 | a |
| IS 13215 | 89.51 | b | IS 13215 | 135.75 | b | IS 11005 | 305.487805 | a | IS 13211 | 70.17 | b | IS 31637 | 7.56 | a | IS 14485 | 21.47 | a | IS 18833 | 34.01 | b |
| IS 40031 | 85.04 | c | IS 18234 | 131.74 | c | IS 13215 | 304.393939 | a | IS 32263 | 69.13 | b | IS 33844 | 6.57 | b | IS 2134 | 21.36 | a | IS 1128 | 33.55 | b |
| IS 18234 | 84.30 | d | IS 40031 | 130.47 | d | IS 40238 | 293.906736 | b | IS 40031 | 65.57 | c | IS 29605 | 6.54 | b | IS 18833 | 21.21 | ab | IS 11005 | 31.29 | bc |
| IS 40238 | 80.96 | e | IS 40238 | 126.91 | e | IS 18833 | 285.645161 | c | IS 18234 | 65.38 | c | IS 25476 | 6.50 | b | IS 13065 | 20.56 | ab | IS 2134 | 30.34 | bcd |
| IS 2153 | 80.24 | e | IS 33844 | 125.99 | ef | IS 2153 | 282.184466 | cd | IS 22428 | 65.23 | c | IS 32263 | 6.41 | bc | IS 13215 | 20.31 | ab | IS 14485 | 30.22 | bcd |
| IS 33844 | 79.26 | f | IS 3399 | 125.44 | f | IS 25476 | 279.244565 | de | IS 13068 | 65.09 | c | IS 40031 | 6.36 | bc | IS 35474 | 19.25 | b | IS 27325 | 28.42 | cde |
| IS 3399 | 78.77 | f | IS 2153 | 124.43 | g | IS 40031 | 275.743243 | ef | IS 29605 | 64.63 | c | IS 29508 | 6.21 | bcd | IS 34283 | 17.63 | c | IS 22428 | 27.43 | cdef |
| IS 18833 | 76.75 | g | IS 21858 | 122.92 | h | IS 35217 | 272.04386 | fg | IS 10897 | 64.63 | c | IS 13215 | 6.14 | bcde | IS 29605 | 17.54 | c | IS 3399 | 26.19 | defg |
| IS 21858 | 75.47 | h | IS 27325 | 122.15 | hi | IS 35474 | 270.32967 | fgh | IS 27325 | 64.51 | c | IS 3399 | 5.98 | cdef | IS 32252 | 17.45 | c | IS 34283 | 25.12 | efgh |
| IS 10897 | 75.36 | h | IS 18833 | 121.69 | ij | IS 27325 | 269.723926 | fgh | IS 2134 | 63.83 | cd | IS 35474 | 5.96 | cdef | IS 14010 | 17.01 | c | IS 2348 | 24.80 | efgh |
| IS 27325 | 74.87 | h | IS 35217 | 120.91 | j | IS 33844 | 267.56338 | gh | IS 14010 | 63.74 | cd | IS 12965 | 5.95 | cdef | IS 22407 | 16.75 | cd | IS 13215 | 24.54 | efgh |
| IS 35217 | 73.99 | i | IS 10897 | 119.72 | k | IS 32252 | 263.784615 | h | IS 13215 | 63.33 | cde | IS 22428 | 5.86 | defg | IS 27325 | 16.55 | cde | IS 35474 | 24.22 | efghi |
| IS 40161 | 73.94 | i | IS 31637 | 119.55 | k | IS 32263 | 255.331034 | i | IS 31637 | 62.94 | cde | IS 14010 | 5.70 | efgh | IS 13211 | 15.72 | cdef | IS 13065 | 24.12 | efghij |
| IS 35474 | 73.33 | i | IS 32263 | 119.48 | kl | IS 22428 | 251.90184 | i | IS 40238 | 61.73 | def | IS 40238 | 5.68 | efgh | IS 13068 | 15.67 | cdef | IS 13068 | 23.96 | efghij |
| IS 22428 | 72.43 | j | IS 13211 | 118.96 | klm | IS 34283 | 245.531915 | j | IS 11005 | 61.05 | efg | IS 35217 | 5.56 | fghi | IS 3399 | 14.73 | def | IS 10897 | 23.60 | efghijk |
| IS 32263 | 72.40 | j | IS 40161 | 118.89 | klm | IS 14485 | 243.6 | j | IS 40161 | 60.84 | efg | IS 2134 | 5.55 | fghi | IS 40031 | 14.53 | ef | IS 33844 | 23.04 | fghijk |
| IS 13211 | 72.29 | j | IS 35474 | 118.79 | klm | IS 10897 | 234.773869 | k | IS 8330 | 60.35 | fgh | IS 13068 | 5.43 | ghij | IS 22606 | 13.62 | fg | IS 29508 | 23.00 | fghijk |
| IS 2134 | 72.23 | j | IS 22428 | 118.63 | klm | IS 18234 | 230.994764 | k | IS 12965 | 59.52 | fghi | IS 13065 | 5.39 | ghij | IS 8330 | 13.60 | fg | IS 35217 | 22.35 | ghijk |
| IS 31637 | 71.81 | j | IS 25476 | 118.03 | lmn | IS 31637 | 225.785714 | l | IS 35474 | 59.35 | fghi | IS 40161 | 5.38 | ghij | IS 10897 | 13.52 | fg | IS 12919 | 22.17 | ghijk |
| IS 11005 | 71.00 | k | IS 2134 | 117.95 | mn | IS 3399 | 225.635838 | l | IS 3399 | 58.79 | ghij | IS 11005 | 5.37 | ghij | IS 29508 | 12.18 | gh | IS 18234 | 22.09 | ghijk |
| IS 25476 | 69.59 | l | IS 11005 | 116.85 | n | IS 13211 | 225.624339 | l | IS 2348 | 57.90 | hijk | IS 21858 | 5.27 | hijk | IS 40161 | 11.45 | hi | IS 2153 | 22.03 | ghijk |
| IS 13068 | 68.38 | m | IS 13068 | 114.51 | o | IS 14010 | 203.325 | m | IS 34283 | 57.76 | hijk | IS 8330 | 5.24 | hijk | IS 25476 | 10.87 | hij | IS 29605 | 21.73 | ghijk |
| IS 2348 | 66.25 | n | IS 14010 | 112.03 | p | IS 29605 | 199.959276 | mn | IS 29508 | 57.56 | ijk | IS 18234 | 5.09 | ijk | IS 2153 | 10.40 | hijk | IS 40031 | 21.38 | ghijk |
| IS 29605 | 66.13 | n | IS 29605 | 111.53 | p | IS 21858 | 195.57754 | no | IS 35217 | 57.39 | ijk | IS 2153 | 5.08 | ijk | IS 11005 | 10.03 | ijk | IS 40238 | 21.01 | ghijk |
| IS 14010 | 65.71 | n | IS 14485 | 110.97 | p | IS 29508 | 193.631579 | o | IS 13065 | 56.76 | ijkl | IS 13211 | 5.02 | jk | IS 2348 | 9.94 | ijk | IS 13211 | 20.14 | hijk |
| IS 14485 | 63.50 | o | IS 2348 | 110.74 | p | IS 2134 | 192.941176 | o | IS 2153 | 56.48 | jklm | IS 2348 | 4.96 | jk | IS 31637 | 9.61 | ijkl | IS 21858 | 19.93 | hijk |
| IS 1128 | 63.18 | o | IS 1128 | 109.63 | q | IS 1128 | 185.852792 | p | IS 21858 | 56.44 | jklm | IS 27325 | 4.95 | jk | IS 18234 | 9.56 | ijkl | IS 31637 | 19.14 | ijkl |
| IS 34283 | 62.26 | p | IS 34283 | 109.44 | q | IS 40161 | 178.85 | q | IS 25476 | 56.21 | jklm | IS 32252 | 4.93 | jk | IS 12919 | 9.37 | ijkl | IS 8330 | 19.08 | ijkl |
| IS 13065 | 59.26 | q | IS 13065 | 106.41 | r | IS 22407 | 156.289855 | r | IS 33844 | 55.46 | klmn | IS 1128 | 4.84 | k | IS 40238 | 9.07 | ijklm | IS 14010 | 18.77 | jklm |
| IS 29508 | 58.44 | r | IS 29508 | 105.92 | r | IS 8330 | 145.363128 | s | IS 1128 | 55.14 | klmn | IS 10897 | 4.83 | k | IS 35217 | 8.67 | jklm | IS 12965 | 18.38 | klm |
| IS 12965 | 58.24 | r | IS 12965 | 103.10 | s | IS 2348 | 143.058824 | s | IS 18833 | 54.35 | lmn | IS 34283 | 4.76 | k | IS 32263 | 8.37 | klm | IS 22407 | 15.34 | lmn |
| IS 22407 | 56.95 | s | IS 22407 | 102.67 | s | IS 13065 | 133.054852 | t | IS 32252 | 53.73 | mn | IS 22407 | 4.09 | l | IS 1128 | 7.34 | lm | IS 32252 | 15.10 | lmn |
| IS 8330 | 55.50 | t | IS 32252 | 100.95 | t | IS 12965 | 125.801136 | u | IS 14485 | 53.25 | n | IS 14485 | 4.05 | l | IS 21858 | 6.97 | m | IS 25476 | 14.56 | mn |
| IS 32252 | 53.65 | u | IS 8330 | 100.51 | t | IS 13068 | 103.64532 | v | IS 22407 | 51.26 | o | IS 18833 | 3.91 | l | IS 33844 | 4.64 | n | IS 40161 | 12.60 | n |
| IS 12919 | 52.64 | v | IS 12919 | 97.22 | u | IS 12919 | 98.6363636 | v | IS 12919 | 39.59 | p | IS 12919 | 3.84 | l | IS 12965 | 2.63 | o | IS 32263 | 11.68 | n |

Table S5. Mean grouping by Student-Newman-Keuls Test of sorghum, pearl millet and pigeonpea for all the quantitative traits recorded

| Acc.No | Panicle width (Cm) | groups | Acc.No | Single Plant Yield (g) | groups | Acc.No | Single Plant Seed Yield (g) | groups |
| --- | --- | --- | --- | --- | --- | --- | --- | --- |
| IS 22606 | 26.60 | a | IS 18234 | 58.08 | a | IS 40238 | 44.59 | a |
| IS 18833 | 22.49 | b | IS 40238 | 57.38 | a | IS 13215 | 43.40 | ab |
| IS 1128 | 19.13 | c | IS 35474 | 57.01 | a | IS 18234 | 42.42 | abc |
| IS 14485 | 18.96 | c | IS 13215 | 56.75 | a | IS 35474 | 41.79 | abc |
| IS 18234 | 13.78 | d | IS 27325 | 53.34 | ab | IS 31637 | 39.02 | abcd |
| IS 13215 | 12.59 | e | IS 33844 | 52.70 | abc | IS 27325 | 38.66 | abcd |
| IS 11005 | 12.03 | e | IS 31637 | 51.86 | abc | IS 33844 | 38.02 | bcd |
| IS 13211 | 11.91 | e | IS 32263 | 50.64 | abcd | IS 40031 | 36.74 | cd |
| IS 2348 | 11.08 | f | IS 2134 | 50.27 | abcd | IS 32263 | 36.57 | cd |
| IS 2153 | 11.02 | f | IS 40031 | 49.03 | abcd | IS 11005 | 35.57 | d |
| IS 34283 | 10.48 | fg | IS 11005 | 46.57 | abcde | IS 35217 | 32.92 | de |
| IS 25476 | 10.10 | gh | IS 22606 | 44.87 | abcdef | IS 21858 | 30.20 | ef |
| IS 13068 | 9.77 | gh | IS 35217 | 44.43 | abcdef | IS 3399 | 26.79 | fg |
| IS 27325 | 9.50 | hi | IS 3399 | 40.18 | abcdefg | IS 22606 | 25.93 | fgh |
| IS 31637 | 9.42 | hi | IS 21858 | 38.79 | abcdefgh | IS 25476 | 25.32 | fgh |
| IS 35474 | 8.72 | ij | IS 25476 | 36.11 | abcdefghi | IS 22428 | 24.99 | fgh |
| IS 2134 | 8.60 | ij | IS 22428 | 35.99 | abcdefghi | IS 14010 | 23.46 | ghi |
| IS 10897 | 8.25 | jk | IS 14010 | 34.00 | bcdefghi | IS 2153 | 20.60 | hij |
| IS 35217 | 8.22 | jk | IS 2153 | 31.92 | bcdefghi | IS 40161 | 19.01 | ijk |
| IS 40238 | 8.21 | jk | IS 2348 | 30.62 | cdefghi | IS 2134 | 18.50 | ijk |
| IS 21858 | 8.09 | jkl | IS 40161 | 29.04 | defghij | IS 2348 | 18.32 | ijk |
| IS 14010 | 8.05 | jkl | IS 10897 | 26.80 | efghij | IS 34283 | 17.77 | ijkl |
| IS 13065 | 7.95 | jkl | IS 34283 | 26.75 | efghij | IS 29508 | 17.66 | ijkl |
| IS 29605 | 7.93 | jkl | IS 29508 | 26.29 | efghij | IS 13211 | 16.97 | jkl |
| IS 33844 | 7.44 | klm | IS 14485 | 25.61 | efghij | IS 8330 | 15.33 | jkl |
| IS 40031 | 7.25 | klmn | IS 13211 | 25.32 | efghij | IS 12965 | 14.84 | jklm |
| IS 29508 | 7.22 | klmn | IS 8330 | 24.74 | efghij | IS 10897 | 14.30 | jklm |
| IS 12965 | 7.00 | lmn | IS 12965 | 23.20 | fghij | IS 14485 | 14.12 | jklm |
| IS 32263 | 6.83 | mno | IS 13068 | 22.80 | fghij | IS 13068 | 13.84 | jklm |
| IS 12919 | 6.47 | mnop | IS 32252 | 22.13 | fghij | IS 32252 | 13.73 | jklm |
| IS 3399 | 6.44 | mnop | IS 29605 | 22.05 | fghij | IS 29605 | 12.86 | klm |
| IS 22407 | 6.33 | mnop | IS 1128 | 20.09 | ghij | IS 1128 | 11.01 | lmn |
| IS 22428 | 6.21 | nop | IS 22407 | 17.60 | ghij | IS 22407 | 8.68 | mn |
| IS 8330 | 5.79 | op | IS 13065 | 16.49 | hij | IS 13065 | 6.76 | no |
| IS 40161 | 5.78 | op | IS 18833 | 14.64 | ij | IS 18833 | 6.17 | no |
| IS 32252 | 5.43 | p | IS 12919 | 6.82 | j | IS 12919 | 1.94 | o |

| Acc.No | Basal Tillers | Groups | Acc.No | Days to flowering | Groups | Acc.No | Leaf length (cm) | Groups | Acc.No | Leaf width (cm) | Groups | Acc.No | Number of leaves | Groups | Acc.No | Panicle length (cm) | Groups |
| --- | --- | --- | --- | --- | --- | --- | --- | --- | --- | --- | --- | --- | --- | --- | --- | --- | --- |
| IP 21752 | 6.88 | a | IP 6037 | 60.39 | a | IP 5253 | 64.05 | a | IP 11984 | 31.80 | a | IP 7468 | 10.52 | a | IP 13112 | 43.45 | a |
| IP 21640 | 5.32 | b | IP 7468 | 60.14 | a | IP 11984 | 63.78 | a | IP 18157 | 31.36 | ab | IP 13363 | 10.28 | ab | IP 11984 | 39.01 | b |
| IP 22039 | 3.96 | c | IP 13363 | 59.87 | ab | IP 3616 | 59.63 | b | IP 13363 | 30.31 | abc | IP 5253 | 10.21 | abc | IP 10705 | 31.54 | c |
| IP 9824 | 2.95 | d | IP 20407 | 59.34 | ab | IP 13112 | 59.41 | b | IP 10705 | 30.04 | abcd | IP 11984 | 10.19 | abc | IP 5253 | 30.63 | c |
| IP 20349 | 2.59 | de | IP 12138 | 58.50 | abc | IP 19434 | 58.48 | bc | IP 17632 | 29.73 | abcd | IP 14418 | 10.15 | abc | IP 5441 | 29.84 | c |
| IP 6244 | 2.36 | ef | IP 9824 | 58.49 | abc | IP 12155 | 58.45 | bc | IP 13112 | 29.15 | abcde | IP 3616 | 10.09 | abc | IP 20407 | 28.57 | cd |
| IP 3269 | 2.26 | efg | IP 5253 | 57.74 | abcd | IP 18157 | 56.89 | bcd | IP 6434 | 28.94 | abcde | IP 5441 | 10.08 | abc | IP 5900 | 28.31 | cd |
| IP 20407 | 2.21 | efgh | IP 14418 | 57.13 | abcde | IP 10705 | 56.35 | bcde | IP 19434 | 28.75 | abcdef | IP 20407 | 10.08 | abc | IP 19434 | 27.99 | cd |
| IP 18147 | 2.21 | efgh | IP 14071 | 56.03 | bcdef | IP 5441 | 56.09 | bcde | IP 3616 | 28.53 | abcdefg | IP 5900 | 10.08 | abc | IP 3389 | 25.65 | de |
| IP 6037 | 2.19 | efghi | IP 5900 | 54.98 | cdef | IP 5900 | 54.84 | bcdef | IP 10085 | 28.20 | bcdefgh | IP 12155 | 10.04 | abc | IP 3616 | 24.00 | ef |
| IP 12138 | 2.00 | efghij | IP 10705 | 54.60 | defg | IP 13363 | 54.55 | bcdef | IP 4952 | 28.13 | bcdefgh | IP 19434 | 9.93 | abc | IP 4952 | 23.95 | ef |
| IP 13459 | 1.90 | efghijk | IP 19434 | 54.35 | defgh | IP 6434 | 54.47 | bcdef | IP 5900 | 27.84 | bcdefgh | IP 6434 | 9.82 | abc | IP 6434 | 23.68 | ef |
| IP 11677 | 1.87 | efghijk | IP 13112 | 54.25 | defgh | IP 10085 | 54.43 | bcdef | IP 9446 | 27.82 | bcdefgh | IP 12138 | 9.80 | abc | IP 12138 | 22.87 | ef |
| IP 5253 | 1.65 | fghijk | IP 11984 | 53.99 | defghi | IP 4952 | 54.33 | bcdef | IP 14071 | 27.75 | bcdefgh | IP 3389 | 9.73 | abc | IP 11577 | 22.71 | ef |
| IP 3616 | 1.62 | fghijk | IP 10085 | 53.41 | efghij | IP 8761 | 53.63 | cdef | IP 8761 | 27.15 | cdefghi | IP 9824 | 9.67 | bcd | IP 6244 | 22.61 | ef |
| IP 4952 | 1.59 | fghijk | IP 12155 | 52.98 | fghijk | IP 6037 | 53.55 | cdef | IP 12155 | 27.12 | cdefghi | IP 13112 | 9.66 | bcd | IP 13363 | 21.93 | efg |
| IP 5900 | 1.56 | ghijk | IP 11577 | 52.76 | fghijk | IP 14418 | 52.96 | cdef | IP 7468 | 26.85 | cdefghi | IP 10085 | 9.60 | bcd | IP 6037 | 21.46 | efg |
| IP 14418 | 1.55 | ghijk | IP 6434 | 52.31 | fghijk | IP 12138 | 52.62 | defg | IP 6037 | 26.76 | cdefghi | IP 6037 | 9.50 | bcde | IP 8761 | 21.30 | efg |
| IP 3389 | 1.46 | ghijk | IP 8761 | 52.24 | fghijk | IP 6244 | 52.52 | defg | IP 5253 | 26.59 | cdefghi | IP 14071 | 9.45 | bcdef | IP 18157 | 20.84 | fgh |
| IP 17632 | 1.45 | hijk | IP 6244 | 51.75 | fghijk | IP 6109 | 51.96 | defg | IP 6109 | 26.37 | cdefghi | IP 10705 | 9.43 | cdef | IP 14418 | 20.10 | fghi |
| IP 8761 | 1.40 | ijk | IP 3616 | 50.49 | ghijkl | IP 7468 | 51.70 | defg | IP 5441 | 26.23 | defghi | IP 18157 | 9.42 | cdef | IP 10085 | 19.94 | fghi |
| IP 9446 | 1.37 | jk | IP 18157 | 50.35 | ghijkl | IP 11577 | 51.43 | defg | IP 11677 | 26.20 | defghi | IP 11577 | 9.37 | cdef | IP 14071 | 19.90 | fghi |
| IP 6109 | 1.36 | jk | IP 5441 | 50.27 | ghijkl | IP 14071 | 51.33 | defg | IP 11577 | 26.16 | defghi | IP 6244 | 8.95 | defg | IP 9824 | 19.85 | fghi |
| IP 14071 | 1.32 | jk | IP 4952 | 50.15 | hijkl | IP 17632 | 51.13 | defg | IP 14418 | 26.07 | defghi | IP 8761 | 8.86 | efgh | IP 7468 | 19.64 | fghi |
| IP 19434 | 1.32 | jk | IP 22039 | 49.89 | ijkl | IP 20407 | 51.08 | defg | IP 6244 | 25.30 | efghi | IP 6109 | 8.80 | efgh | IP 13459 | 17.81 | ghij |
| IP 6434 | 1.31 | jk | IP 3389 | 49.60 | jkl | IP 11677 | 50.87 | defg | IP 12138 | 24.84 | fghi | IP 17632 | 8.75 | fgh | IP 12155 | 17.74 | ghij |
| IP 11577 | 1.31 | jk | IP 11677 | 48.76 | klm | IP 20349 | 50.82 | defg | IP 9824 | 24.68 | ghi | IP 13459 | 8.68 | gh | IP 20349 | 17.64 | ghij |
| IP 12155 | 1.26 | jk | IP 6109 | 46.61 | lmn | IP 3389 | 50.63 | efg | IP 13459 | 24.60 | ghi | IP 11677 | 8.60 | ghi | IP 17632 | 17.59 | ghij |
| IP 10705 | 1.25 | jk | IP 20349 | 46.16 | mn | IP 9446 | 49.03 | fg | IP 3389 | 24.20 | hi | IP 20349 | 8.59 | ghi | IP 9446 | 17.16 | ghij |
| IP 5441 | 1.25 | jk | IP 13459 | 45.01 | no | IP 13459 | 46.95 | g | IP 20407 | 23.27 | i | IP 4952 | 8.50 | ghi | IP 11677 | 16.42 | hij |
| IP 13112 | 1.23 | jk | IP 21640 | 44.72 | no | IP 9824 | 46.84 | g | IP 22039 | 20.67 | j | IP 22039 | 8.31 | ghi | IP 3269 | 16.06 | hij |
| IP 7468 | 1.19 | jk | IP 21752 | 42.24 | op | IP 3269 | 41.62 | h | IP 20349 | 20.41 | j | IP 21640 | 8.13 | hi | IP 6109 | 15.73 | ij |
| IP 10085 | 1.16 | jk | IP 3269 | 42.18 | op | IP 22039 | 37.90 | i | IP 3269 | 18.28 | j | IP 9446 | 7.97 | i | IP 18147 | 13.65 | jk |
| IP 13363 | 1.13 | jk | IP 17632 | 40.25 | p | IP 18147 | 36.98 | i | IP 18147 | 17.94 | j | IP 3269 | 7.48 | j | IP 22039 | 11.21 | kl |
| IP 18157 | 1.07 | k | IP 9446 | 39.99 | p | IP 21640 | 31.36 | j | IP 21640 | 13.83 | k | IP 21752 | 7.30 | j | IP 21752 | 10.54 | kl |
| IP 11984 | 1.04 | k | IP 18147 | 39.29 | p | IP 21752 | 27.97 | k | IP 21752 | 12.84 | k | IP 18147 | 7.21 | j | IP 21640 | 9.62 | l |

| Acc.No | Panicle width (cm) | Groups | Acc.No | Plant height (cm) | Groups | Acc.No | Stem thickness (mm) | Groups |
| --- | --- | --- | --- | --- | --- | --- | --- | --- |
| IP 13363 | 31.13 | a | IP 13363 | 208.82 | a | IP 11984 | 10.53 | a |
| IP 14071 | 28.61 | b | IP 14418 | 207.54 | a | IP 13363 | 9.13 | b |
| IP 17632 | 25.73 | c | IP 12155 | 199.14 | ab | IP 14071 | 8.99 | b |
| IP 18157 | 24.79 | cd | IP 3616 | 195.85 | abc | IP 10705 | 8.95 | b |
| IP 9446 | 24.26 | cde | IP 13112 | 195.31 | abcd | IP 7468 | 8.93 | b |
| IP 8761 | 24.05 | cdef | IP 10705 | 188.60 | bcde | IP 13112 | 8.81 | bc |
| IP 6434 | 23.88 | cdef | IP 6037 | 188.32 | bcde | IP 5253 | 8.68 | bcd |
| IP 5253 | 22.92 | cdefg | IP 6434 | 187.92 | bcde | IP 6434 | 8.62 | bcde |
| IP 7468 | 22.55 | defgh | IP 19434 | 187.79 | bcde | IP 18157 | 8.62 | bcde |
| IP 10085 | 22.47 | defgh | IP 5253 | 186.44 | bcde | IP 4952 | 8.40 | bcde |
| IP 6109 | 22.32 | defgh | IP 12138 | 185.97 | bcde | IP 19434 | 8.37 | bcde |
| IP 4952 | 22.05 | defgh | IP 11984 | 181.43 | bcdef | IP 5441 | 8.31 | bcdef |
| IP 11984 | 21.61 | defghi | IP 5441 | 181.24 | bcdef | IP 5900 | 8.23 | bcdef |
| IP 5441 | 21.39 | efghij | IP 7468 | 181.07 | bcdef | IP 3616 | 8.07 | bcdef |
| IP 3616 | 20.97 | fghij | IP 3389 | 180.82 | bcdef | IP 14418 | 8.06 | bcdef |
| IP 19434 | 20.58 | ghijk | IP 5900 | 179.76 | bcdefg | IP 12155 | 8.02 | bcdefg |
| IP 12155 | 20.22 | ghijkl | IP 11677 | 177.79 | cdefg | IP 8761 | 8.01 | bcdefg |
| IP 3389 | 19.72 | ghijkl | IP 20349 | 176.32 | cdefg | IP 6244 | 7.70 | cdefgh |
| IP 6244 | 19.68 | ghijkl | IP 4952 | 175.14 | cdefg | IP 6109 | 7.63 | cdefgh |
| IP 5900 | 19.66 | ghijkl | IP 8761 | 174.12 | defgh | IP 10085 | 7.57 | defgh |
| IP 13459 | 19.57 | ghijkl | IP 20407 | 173.88 | defgh | IP 3389 | 7.55 | defgh |
| IP 11577 | 19.49 | ghijkl | IP 9824 | 173.45 | efgh | IP 9446 | 7.54 | defgh |
| IP 10705 | 19.41 | hijkl | IP 6109 | 173.12 | efgh | IP 6037 | 7.52 | defgh |
| IP 14418 | 19.25 | hijkl | IP 11577 | 172.66 | efgh | IP 20407 | 7.47 | defgh |
| IP 13112 | 18.59 | ijkl | IP 17632 | 171.49 | efgh | IP 12138 | 7.47 | defgh |
| IP 6037 | 18.56 | ijkl | IP 18157 | 169.70 | efghi | IP 17632 | 7.37 | efgh |
| IP 9824 | 18.21 | ijkl | IP 9446 | 160.43 | fghij | IP 11577 | 7.09 | fghi |
| IP 11677 | 18.03 | jkl | IP 10085 | 159.13 | ghij | IP 11677 | 6.85 | ghi |
| IP 12138 | 17.45 | kl | IP 14071 | 158.84 | ghij | IP 13459 | 6.82 | ghi |
| IP 20349 | 16.90 | lm | IP 13459 | 154.62 | hij | IP 20349 | 6.68 | hi |
| IP 20407 | 16.84 | lm | IP 6244 | 152.45 | ij | IP 9824 | 6.59 | hi |
| IP 18147 | 14.81 | m | IP 22039 | 150.98 | j | IP 22039 | 6.15 | ij |
| IP 3269 | 14.71 | m | IP 3269 | 133.65 | k | IP 18147 | 5.74 | jk |
| IP 21752 | 11.94 | n | IP 21640 | 131.60 | k | IP 3269 | 5.52 | jkl |
| IP 22039 | 11.66 | n | IP 18147 | 124.05 | k | IP 21752 | 5.03 | kl |
| IP 21640 | 9.64 | n | IP 21752 | 123.14 | k | IP 21640 | 4.69 | l |

| Acc.No | Leaflet length (cm) | Groups | Acc.No | Leaflet width (cm) | Groups | Acc.No | Plant height (cm) | Groups | Acc.No | Primary branches per plant | Groups | Acc.No | Secondary branches per plant | Groups |
| --- | --- | --- | --- | --- | --- | --- | --- | --- | --- | --- | --- | --- | --- | --- |
| ICP 9150 | 11.17 | a | ICP 9150 | 5.11 | a | ICP 13545 | 210.00 | a | ICP 7057 | 21.07 | a | ICP 7057 | 11.57 | a |
| ICP 13545 | 9.71 | b | ICP 13415 | 3.85 | b | ICP 14388 | 201.80 | ab | ICP 13545 | 18.00 | b | ICP 12189 | 7.04 | b |
| ICP 13415 | 9.11 | bc | ICP 13545 | 3.66 | b | ICP 13316 | 201.30 | ab | ICP 12190 | 14.81 | c | ICP 10880 | 6.32 | bc |
| ICP 13316 | 8.90 | cd | ICP 13828 | 3.33 | c | ICP 7057 | 190.00 | bc | ICP 11475 | 13.57 | cd | ICP 7621 | 5.53 | bc |
| ICP 14388 | 8.72 | cde | ICP 14059 | 3.21 | cd | ICP 15148 | 186.00 | bcd | ICP 10889 | 12.25 | cde | ICP 12190 | 5.50 | bc |
| ICP 13828 | 8.53 | cdef | ICP 2309 | 3.14 | cd | ICP 12840 | 177.63 | cde | ICP 10880 | 12.18 | cde | ICP 13545 | 5.00 | bc |
| ICP 9877 | 8.16 | defg | ICP 14169 | 3.13 | cd | ICP 13415 | 176.41 | cde | ICP 7621 | 11.84 | cde | ICP 13546 | 4.83 | bc |
| ICP 15148 | 8.16 | defg | ICP 9877 | 3.11 | cd | ICP 13999 | 174.17 | cdef | ICP 14388 | 11.08 | cdef | ICP 6399 | 4.37 | bc |
| ICP 14169 | 8.14 | defg | ICP 14388 | 3.07 | cd | ICP 9877 | 173.04 | cdef | ICP 11480 | 10.63 | cdef | ICP 15148 | 3.90 | bc |
| ICP 2309 | 8.14 | defg | ICP 13316 | 3.06 | cd | ICP 10889 | 168.13 | cdefg | ICP 12840 | 10.21 | defg | ICP 16344 | 3.77 | bc |
| ICP 12041 | 8.01 | defg | ICP 12041 | 2.98 | cde | ICP 12190 | 166.73 | defg | ICP 11491 | 10.13 | defg | ICP 2309 | 3.76 | bc |
| ICP 14059 | 7.81 | efgh | ICP 15148 | 2.96 | cde | ICP 10880 | 162.86 | efg | ICP 13546 | 10.04 | defg | ICP 10889 | 3.13 | bc |
| ICP 12840 | 7.61 | fghi | ICP 13999 | 2.84 | def | ICP 9150 | 158.89 | efgh | ICP 12189 | 10.04 | defg | ICP 13999 | 3.07 | bc |
| ICP 14233 | 7.34 | ghij | ICP 14233 | 2.83 | def | ICP 12189 | 156.43 | efgh | ICP 15148 | 9.90 | defg | ICP 11491 | 2.80 | bc |
| ICP 13999 | 7.33 | ghij | ICP 7057 | 2.62 | efg | ICP 13628 | 154.82 | efghi | ICP 13999 | 9.83 | defg | ICP 13316 | 2.78 | bc |
| ICP 7057 | 7.24 | ghijk | ICP 10889 | 2.62 | efg | ICP 14059 | 151.52 | fghij | ICP 13316 | 9.83 | defg | ICP 13889 | 2.56 | bc |
| ICP 10880 | 7.04 | hijkl | ICP 13628 | 2.62 | efg | ICP 12041 | 148.15 | ghij | ICP 16344 | 9.52 | defgh | ICP 14169 | 2.35 | bc |
| ICP 10889 | 6.99 | hijkl | ICP 12840 | 2.62 | efg | ICP 14169 | 144.71 | ghijk | ICP 6399 | 9.48 | defgh | ICP 11475 | 2.30 | bc |
| ICP 13628 | 6.87 | hijklm | ICP 11480 | 2.50 | fgh | ICP 13575 | 138.10 | hijkl | ICP 13828 | 9.46 | defgh | ICP 12840 | 2.16 | bc |
| ICP 12190 | 6.73 | ijklm | ICP 13889 | 2.47 | fgh | ICP 14233 | 138.00 | hijkl | ICP 2309 | 9.24 | defgh | ICP 13828 | 1.88 | bc |
| ICP 12189 | 6.71 | ijklm | ICP 12189 | 2.46 | fgh | ICP 13828 | 137.80 | hijkl | ICP 13575 | 9.00 | defgh | ICP 12041 | 1.70 | bc |
| ICP 13546 | 6.61 | ijklm | ICP 10880 | 2.45 | fgh | ICP 13546 | 132.17 | ijklm | ICP 9122 | 9.00 | defgh | ICP 13628 | 1.64 | bc |
| ICP 6399 | 6.57 | ijklm | ICP 13546 | 2.44 | fgh | ICP 7621 | 131.84 | ijklm | ICP 11485 | 8.39 | efgh | ICP 13575 | 1.41 | bc |
| ICP 13889 | 6.54 | ijklm | ICP 7621 | 2.44 | fgh | ICP 6399 | 131.67 | ijklm | ICP 14059 | 8.09 | efgh | ICP 13415 | 1.31 | bc |
| ICP 7035 | 6.51 | jklm | ICP 13575 | 2.42 | fgh | ICP 11475 | 130.00 | jklm | ICP 14169 | 7.85 | efgh | ICP 14059 | 1.18 | bc |
| ICP 7621 | 6.43 | jklmn | ICP 16344 | 2.40 | fgh | ICP 13889 | 129.72 | jklm | ICP 9124 | 7.69 | efgh | ICP 14233 | 1.10 | bc |
| ICP 13575 | 6.38 | jklmn | ICP 7035 | 2.39 | fgh | ICP 16344 | 127.58 | jklm | ICP 14233 | 7.65 | efgh | ICP 14296 | 1.07 | bc |
| ICP 14296 | 6.34 | jklmn | ICP 6399 | 2.37 | gh | ICP 2309 | 127.38 | jklm | ICP 13889 | 7.61 | efgh | ICP 9124 | 1.03 | bc |
| ICP 16344 | 6.20 | klmn | ICP 11485 | 2.36 | gh | ICP 11491 | 120.67 | klmn | ICP 13628 | 7.06 | efgh | ICP 11480 | 0.93 | bc |
| ICP 9124 | 6.18 | klmn | ICP 12190 | 2.35 | gh | ICP 14296 | 115.89 | lmno | ICP 13415 | 6.31 | fgh | ICP 9877 | 0.86 | bc |
| ICP 11480 | 6.11 | lmn | ICP 14296 | 2.32 | gh | ICP 11480 | 113.70 | lmno | ICP 12041 | 6.15 | fgh | ICP 11485 | 0.83 | bc |
| ICP 11475 | 6.06 | lmn | ICP 11491 | 2.27 | gh | ICP 9122 | 111.46 | mno | ICP 7035 | 6.00 | fgh | ICP 14388 | 0.80 | bc |
| ICP 11491 | 5.79 | mn | ICP 11475 | 2.20 | gh | ICP 7035 | 101.52 | no | ICP 14296 | 5.75 | fgh | ICP 9122 | 0.71 | bc |
| ICP 9122 | 5.74 | mn | ICP 9124 | 2.15 | gh | ICP 9124 | 99.48 | no | ICP 9877 | 5.04 | gh | ICP 7035 | 0.67 | c |
| ICP 11485 | 5.41 | n | ICP 9122 | 2.05 | h | ICP 11485 | 95.00 | o | ICP 9150 | 4.44 | h | ICP 9150 | 0.26 | c |

| Acc.No | Tertiary branches per plant | Groups | Acc.No | Days to flowering | Groups | Acc.No | Racemes per plant | Groups | Acc.No | Days to 75% maturity | Groups | Acc.No | Pod bearing length (cm) | Groups |
| --- | --- | --- | --- | --- | --- | --- | --- | --- | --- | --- | --- | --- | --- | --- |
| ICP 13999 | 0.90 | a | ICP 13545 | 139.50 | a | ICP 7057 | 115.93 | a | ICP 13545 | 190.00 | a | ICP 13316 | 91.09 | a |
| ICP 10880 | 0.57 | a | ICP 7057 | 137.64 | a | ICP 13545 | 81.00 | b | ICP 7057 | 187.07 | ab | ICP 7057 | 91.07 | a |
| ICP 7621 | 0.47 | a | ICP 14388 | 136.44 | a | ICP 2309 | 67.52 | bc | ICP 14388 | 185.56 | ab | ICP 13545 | 80.00 | ab |
| ICP 12189 | 0.46 | a | ICP 15148 | 131.05 | ab | ICP 10880 | 62.50 | bcd | ICP 15148 | 183.45 | abc | ICP 14388 | 78.00 | abc |
| ICP 7057 | 0.43 | a | ICP 13316 | 126.09 | bc | ICP 7621 | 61.79 | bcd | ICP 13316 | 181.00 | abc | ICP 14059 | 76.85 | abc |
| ICP 16344 | 0.29 | a | ICP 10889 | 126.00 | bc | ICP 13575 | 60.24 | bcde | ICP 13415 | 178.19 | bcd | ICP 12840 | 73.68 | bcd |
| ICP 14233 | 0.20 | a | ICP 10880 | 117.93 | cd | ICP 12190 | 58.50 | bcdef | ICP 10880 | 173.86 | cde | ICP 15148 | 73.25 | bcd |
| ICP 13828 | 0.12 | a | ICP 12190 | 116.85 | cde | ICP 6399 | 53.37 | cdefg | ICP 10889 | 173.50 | cde | ICP 10880 | 72.86 | bcd |
| ICP 14169 | 0.12 | a | ICP 9150 | 115.63 | def | ICP 11491 | 53.33 | cdefg | ICP 9150 | 172.63 | cde | ICP 9877 | 71.43 | bcde |
| ICP 10889 | 0.00 | a | ICP 13415 | 114.31 | defg | ICP 16344 | 51.06 | cdefg | ICP 12190 | 168.96 | def | ICP 13628 | 69.52 | bcde |
| ICP 11475 | 0.00 | a | ICP 9877 | 112.29 | defgh | ICP 12189 | 50.25 | cdefg | ICP 9877 | 168.75 | def | ICP 7621 | 67.63 | bcde |
| ICP 11480 | 0.00 | a | ICP 12840 | 110.05 | defghi | ICP 11475 | 46.17 | cdefgh | ICP 13999 | 168.73 | def | ICP 13999 | 67.17 | bcde |
| ICP 11485 | 0.00 | a | ICP 13999 | 108.37 | defghij | ICP 15148 | 45.45 | cdefgh | ICP 14059 | 167.45 | defg | ICP 12189 | 67.14 | bcde |
| ICP 11491 | 0.00 | a | ICP 11475 | 107.87 | defghijk | ICP 13999 | 44.00 | cdefgh | ICP 12840 | 166.84 | defg | ICP 2309 | 65.71 | bcdef |
| ICP 12041 | 0.00 | a | ICP 14169 | 106.09 | efghijkl | ICP 12840 | 43.05 | cdefgh | ICP 13828 | 165.78 | efgh | ICP 13575 | 65.69 | bcdef |
| ICP 12190 | 0.00 | a | ICP 13546 | 104.35 | fghijklm | ICP 11485 | 42.43 | cdefgh | ICP 14169 | 164.94 | efghi | ICP 12190 | 65.58 | bcdef |
| ICP 12840 | 0.00 | a | ICP 12189 | 104.07 | fghijklm | ICP 10889 | 42.38 | cdefgh | ICP 12189 | 163.68 | efghij | ICP 11475 | 65.43 | bcdef |
| ICP 13316 | 0.00 | a | ICP 14059 | 103.61 | ghijklm | ICP 14388 | 42.20 | cdefgh | ICP 12041 | 162.78 | efghijk | ICP 6399 | 65.19 | bcdef |
| ICP 13415 | 0.00 | a | ICP 2309 | 101.90 | hijklmn | ICP 13546 | 41.04 | cdefgh | ICP 11475 | 162.35 | efghijk | ICP 10889 | 63.75 | bcdef |
| ICP 13545 | 0.00 | a | ICP 13628 | 100.45 | hijklmn | ICP 13316 | 40.57 | cdefgh | ICP 13546 | 159.87 | fghijkl | ICP 13828 | 63.41 | bcdef |
| ICP 13546 | 0.00 | a | ICP 12041 | 99.44 | ijklmn | ICP 14233 | 40.15 | cdefgh | ICP 14233 | 158.20 | fghijklm | ICP 16344 | 62.90 | bcdef |
| ICP 13575 | 0.00 | a | ICP 7621 | 99.37 | ijklmn | ICP 11480 | 37.52 | defgh | ICP 2309 | 157.48 | fghijklm | ICP 13415 | 62.81 | bcdef |
| ICP 13628 | 0.00 | a | ICP 13889 | 99.06 | ijklmn | ICP 13889 | 36.00 | defgh | ICP 13575 | 156.34 | fghijklm | ICP 9150 | 62.04 | bcdef |
| ICP 13889 | 0.00 | a | ICP 6399 | 97.52 | ijklmno | ICP 14169 | 35.38 | defgh | ICP 13628 | 155.09 | ghijklmn | ICP 12041 | 59.07 | bcdefg |
| ICP 14059 | 0.00 | a | ICP 14233 | 97.00 | jklmno | ICP 9122 | 35.21 | defgh | ICP 14296 | 153.93 | hijklmn | ICP 11485 | 58.04 | cdefg |
| ICP 14296 | 0.00 | a | ICP 9124 | 96.52 | jklmnop | ICP 14059 | 33.30 | defgh | ICP 9124 | 153.00 | ijklmn | ICP 13889 | 57.78 | cdefg |
| ICP 14388 | 0.00 | a | ICP 13575 | 95.31 | klmnop | ICP 14296 | 31.82 | efgh | ICP 16344 | 151.55 | jklmn | ICP 14233 | 57.75 | cdefg |
| ICP 15148 | 0.00 | a | ICP 11480 | 95.19 | klmnop | ICP 9150 | 30.81 | fgh | ICP 11480 | 151.30 | jklmn | ICP 14169 | 57.21 | cdefg |
| ICP 2309 | 0.00 | a | ICP 14296 | 95.07 | klmnop | ICP 13628 | 30.21 | fgh | ICP 13889 | 150.39 | klmn | ICP 13546 | 54.57 | defg |
| ICP 6399 | 0.00 | a | ICP 16344 | 94.45 | lmnop | ICP 9124 | 30.00 | fgh | ICP 6399 | 150.00 | klmn | ICP 11491 | 54.17 | defg |
| ICP 7035 | 0.00 | a | ICP 13828 | 91.88 | mnop | ICP 9877 | 28.96 | fgh | ICP 7621 | 148.05 | lmn | ICP 11480 | 50.93 | efg |
| ICP 9122 | 0.00 | a | ICP 11491 | 89.37 | nop | ICP 13415 | 26.91 | gh | ICP 11491 | 145.73 | mn | ICP 14296 | 50.71 | efg |
| ICP 9124 | 0.00 | a | ICP 9122 | 86.79 | opq | ICP 12041 | 23.93 | gh | ICP 9122 | 142.96 | no | ICP 9122 | 50.42 | efg |
| ICP 9150 | 0.00 | a | ICP 11485 | 84.78 | pq | ICP 13828 | 17.66 | h | ICP 11485 | 136.48 | op | ICP 9124 | 44.83 | fg |
| ICP 9877 | 0.00 | a | ICP 7035 | 77.91 | q | ICP 7035 | 17.30 | h | ICP 7035 | 130.45 | p | ICP 7035 | 40.00 | g |

| Acc.No | Pod bearing length (cm) | Groups | Acc.No | Pods per plant | Groups | Acc.No | Seeds per pod | Groups | Acc.No | 100 seed weight (g) | Groups |
| --- | --- | --- | --- | --- | --- | --- | --- | --- | --- | --- | --- |
| ICP 13828 | 8.73 | a | ICP 7057 | 265.29 | a | ICP 13828 | 5.21 | a | ICP 7035 | 22.82 | a |
| ICP 13415 | 7.72 | b | ICP 13545 | 223.00 | b | ICP 9150 | 5.09 | a | ICP 13415 | 16.42 | b |
| ICP 9124 | 7.41 | bc | ICP 2309 | 145.76 | c | ICP 13889 | 4.10 | b | ICP 9150 | 14.71 | c |
| ICP 13889 | 7.41 | bc | ICP 6399 | 125.41 | cd | ICP 9124 | 4.06 | b | ICP 13828 | 14.25 | c |
| ICP 7035 | 7.33 | bc | ICP 12190 | 120.96 | cde | ICP 14059 | 4.02 | bc | ICP 13889 | 13.18 | d |
| ICP 14059 | 7.13 | bc | ICP 7621 | 116.37 | cdef | ICP 14233 | 4.00 | bcd | ICP 9877 | 12.93 | de |
| ICP 9150 | 6.98 | c | ICP 10880 | 116.25 | cdef | ICP 7035 | 3.94 | bcde | ICP 13999 | 12.57 | def |
| ICP 12041 | 6.81 | cd | ICP 13575 | 115.97 | cdef | ICP 13999 | 3.81 | bcdef | ICP 14169 | 12.50 | def |
| ICP 13546 | 6.40 | de | ICP 11480 | 99.63 | cdefg | ICP 13546 | 3.80 | bcdefg | ICP 14296 | 12.41 | def |
| ICP 14169 | 6.29 | def | ICP 16344 | 95.74 | defg | ICP 14169 | 3.79 | bcdefg | ICP 13546 | 11.69 | defg |
| ICP 14233 | 6.08 | efg | ICP 12189 | 94.54 | defg | ICP 13316 | 3.78 | bcdefg | ICP 13628 | 11.57 | efg |
| ICP 9877 | 5.91 | efgh | ICP 11491 | 92.53 | defg | ICP 9877 | 3.71 | bcdefg | ICP 14059 | 11.46 | efg |
| ICP 13628 | 5.82 | efghi | ICP 15148 | 89.80 | defg | ICP 12840 | 3.71 | bcdefg | ICP 12041 | 11.43 | efg |
| ICP 13316 | 5.71 | fghij | ICP 14388 | 87.52 | defg | ICP 13415 | 3.70 | bcdefg | ICP 14233 | 11.23 | fg |
| ICP 12190 | 5.61 | ghijk | ICP 13999 | 84.50 | defg | ICP 13545 | 3.70 | bcdefg | ICP 9124 | 10.47 | gh |
| ICP 12840 | 5.51 | ghijk | ICP 11475 | 80.74 | defgh | ICP 12190 | 3.65 | bcdefgh | ICP 13316 | 10.30 | ghi |
| ICP 12189 | 5.47 | ghijkl | ICP 11485 | 80.09 | defgh | ICP 14388 | 3.62 | bcdefgh | ICP 9122 | 10.18 | ghi |
| ICP 14296 | 5.33 | hijklm | ICP 12840 | 78.68 | defgh | ICP 7621 | 3.60 | bcdefgh | ICP 12189 | 9.67 | hij |
| ICP 14388 | 5.26 | hijklm | ICP 13546 | 75.87 | defgh | ICP 12041 | 3.60 | bcdefgh | ICP 7621 | 9.37 | hijk |
| ICP 16344 | 5.24 | hijklm | ICP 13889 | 66.33 | efgh | ICP 13628 | 3.59 | bcdefgh | ICP 14388 | 9.15 | hijk |
| ICP 11491 | 5.21 | hijklm | ICP 9877 | 65.96 | efgh | ICP 10889 | 3.56 | bcdefgh | ICP 6399 | 9.01 | hijkl |
| ICP 13575 | 5.15 | ijklmn | ICP 13316 | 64.83 | efgh | ICP 10880 | 3.51 | bcdefgh | ICP 15148 | 9.01 | hijkl |
| ICP 11480 | 5.11 | ijklmn | ICP 14233 | 63.05 | fgh | ICP 16344 | 3.49 | bcdefghi | ICP 12190 | 8.75 | ijkl |
| ICP 6399 | 5.09 | ijklmn | ICP 10889 | 62.75 | fgh | ICP 15148 | 3.48 | bcdefghi | ICP 12840 | 8.47 | jkl |
| ICP 13999 | 5.08 | ijklmn | ICP 14059 | 58.52 | gh | ICP 12189 | 3.47 | bcdefghi | ICP 13575 | 8.14 | jkl |
| ICP 10889 | 4.99 | jklmn | ICP 14169 | 57.82 | gh | ICP 11475 | 3.42 | cdefghi | ICP 7057 | 8.11 | jkl |
| ICP 15148 | 4.98 | jklmn | ICP 13415 | 57.50 | gh | ICP 2309 | 3.39 | defghi | ICP 11491 | 8.11 | jkl |
| ICP 10880 | 4.94 | klmn | ICP 9150 | 56.78 | gh | ICP 6399 | 3.33 | efghi | ICP 16344 | 7.99 | jkl |
| ICP 13545 | 4.93 | klmn | ICP 9122 | 55.63 | gh | ICP 14296 | 3.29 | fghi | ICP 10889 | 7.83 | kl |
| ICP 11485 | 4.88 | klmn | ICP 13628 | 51.21 | gh | ICP 7057 | 3.29 | fghi | ICP 2309 | 7.79 | kl |
| ICP 2309 | 4.83 | klmn | ICP 14296 | 50.39 | gh | ICP 11491 | 3.29 | fghi | ICP 11480 | 7.61 | kl |
| ICP 7621 | 4.82 | klmn | ICP 9124 | 47.38 | gh | ICP 11480 | 3.28 | fghi | ICP 13545 | 7.35 | lm |
| ICP 11475 | 4.71 | lmn | ICP 12041 | 40.89 | gh | ICP 13575 | 3.16 | ghi | ICP 11475 | 7.29 | lm |
| ICP 9122 | 4.61 | mn | ICP 13828 | 26.27 | h | ICP 9122 | 3.07 | hi | ICP 10880 | 7.25 | lm |
| ICP 7057 | 4.40 | n | ICP 7035 | 24.64 | h | ICP 11485 | 2.92 | i | ICP 11485 | 6.07 | m |

| Acc.No | Seed yield per plant (cm) | Groups | Acc.No | Dry plant weight (cm) | Groups | Acc.No | Shelling percentage (cm) | Groups | Acc.No | Harvest index (cm) | Groups |
| --- | --- | --- | --- | --- | --- | --- | --- | --- | --- | --- | --- |
| ICP 7057 | 46.21 | a | ICP 13545 | 193.00 | a | ICP 11491 | 67.48 | a | ICP 11491 | 34.00 | a |
| ICP 13545 | 43.00 | a | ICP 7057 | 186.46 | a | ICP 13999 | 67.37 | a | ICP 11480 | 33.50 | ab |
| ICP 12190 | 29.81 | b | ICP 14388 | 104.71 | b | ICP 11480 | 66.87 | a | ICP 11485 | 33.09 | ab |
| ICP 9150 | 28.04 | bc | ICP 12190 | 103.52 | b | ICP 6399 | 65.56 | ab | ICP 7035 | 32.30 | abc |
| ICP 13999 | 27.67 | bc | ICP 13415 | 101.48 | b | ICP 7035 | 64.68 | abc | ICP 6399 | 32.28 | abc |
| ICP 6399 | 26.37 | bc | ICP 13316 | 101.41 | b | ICP 9150 | 63.02 | abcd | ICP 13889 | 31.21 | abc |
| ICP 7621 | 26.11 | bcd | ICP 9150 | 100.88 | b | ICP 2309 | 62.34 | abcde | ICP 2309 | 30.60 | abc |
| ICP 2309 | 25.24 | bcd | ICP 13999 | 96.44 | bc | ICP 11485 | 61.92 | abcde | ICP 9124 | 30.14 | abc |
| ICP 13889 | 24.22 | bcde | ICP 7621 | 94.83 | bc | ICP 13628 | 61.39 | abcde | ICP 9150 | 29.92 | abc |
| ICP 12189 | 21.54 | bcdef | ICP 15148 | 93.50 | bc | ICP 7057 | 60.66 | abcdef | ICP 9877 | 29.20 | abc |
| ICP 13546 | 20.39 | bcdef | ICP 10880 | 91.11 | bc | ICP 12189 | 60.40 | abcdef | ICP 13999 | 28.91 | abc |
| ICP 13415 | 19.72 | bcdef | ICP 12840 | 88.61 | bc | ICP 12190 | 60.24 | abcdef | ICP 16344 | 28.84 | abc |
| ICP 9877 | 19.68 | bcdef | ICP 2309 | 84.99 | bc | ICP 14233 | 60.07 | abcdef | ICP 13575 | 28.52 | abc |
| ICP 11491 | 19.37 | bcdef | ICP 12189 | 82.74 | bc | ICP 9877 | 59.47 | abcdef | ICP 9122 | 28.09 | abc |
| ICP 11480 | 19.15 | bcdef | ICP 6399 | 80.48 | bcd | ICP 14296 | 59.43 | abcdef | ICP 12190 | 27.42 | abcd |
| ICP 14388 | 18.40 | bcdef | ICP 13546 | 78.72 | bcd | ICP 16344 | 58.53 | abcdefg | ICP 7621 | 27.37 | abcd |
| ICP 13575 | 18.21 | bcdef | ICP 13889 | 77.14 | bcd | ICP 14169 | 58.48 | abcdefg | ICP 14296 | 26.87 | abcde |
| ICP 14169 | 18.12 | bcdef | ICP 14169 | 74.17 | bcd | ICP 13889 | 58.31 | abcdefg | ICP 13546 | 25.83 | abcdef |
| ICP 12840 | 18.00 | bcdef | ICP 14059 | 71.62 | bcd | ICP 7621 | 57.97 | abcdefg | ICP 12189 | 25.58 | abcdef |
| ICP 10880 | 17.46 | bcdef | ICP 13575 | 66.87 | bcd | ICP 9122 | 57.67 | abcdefg | ICP 14233 | 25.25 | abcdef |
| ICP 14059 | 17.27 | bcdef | ICP 9877 | 66.51 | bcd | ICP 13415 | 57.41 | abcdefg | ICP 13628 | 24.96 | abcdef |
| ICP 16344 | 17.06 | bcdef | ICP 12041 | 62.99 | bcd | ICP 11475 | 57.12 | abcdefg | ICP 14169 | 24.81 | abcdef |
| ICP 7035 | 16.39 | bcdef | ICP 16344 | 62.86 | bcd | ICP 13575 | 57.00 | abcdefg | ICP 14059 | 24.28 | abcdef |
| ICP 15148 | 16.25 | bcdef | ICP 10889 | 62.83 | bcd | ICP 12840 | 55.72 | bcdefgh | ICP 7057 | 24.17 | bcdef |
| ICP 9124 | 15.17 | cdef | ICP 11475 | 62.45 | bcd | ICP 13545 | 55.45 | bcdefgh | ICP 11475 | 23.90 | bcdef |
| ICP 14233 | 14.80 | cdef | ICP 14233 | 61.91 | bcd | ICP 9124 | 54.95 | cdefgh | ICP 12840 | 22.84 | cdef |
| ICP 11475 | 14.26 | cdef | ICP 11480 | 57.83 | bcd | ICP 14059 | 54.51 | cdefgh | ICP 13415 | 22.53 | cdef |
| ICP 13628 | 14.09 | cdef | ICP 13828 | 57.80 | bcd | ICP 15148 | 54.48 | cdefgh | ICP 10880 | 18.76 | defg |
| ICP 14296 | 14.00 | cdef | ICP 11491 | 55.49 | bcd | ICP 13546 | 54.26 | cdefgh | ICP 15148 | 18.37 | defg |
| ICP 13316 | 13.52 | cdef | ICP 13628 | 54.65 | bcd | ICP 14388 | 52.45 | defgh | ICP 12041 | 17.91 | efg |
| ICP 9122 | 11.58 | def | ICP 9124 | 53.52 | bcd | ICP 13316 | 52.18 | efgh | ICP 13545 | 17.23 | fg |
| ICP 10889 | 10.13 | ef | ICP 7035 | 52.78 | bcd | ICP 12041 | 50.41 | fgh | ICP 14388 | 16.96 | fg |
| ICP 13828 | 9.68 | ef | ICP 14296 | 50.03 | bcd | ICP 10880 | 50.07 | fgh | ICP 13828 | 16.78 | fg |
| ICP 11485 | 9.57 | ef | ICP 9122 | 46.54 | cd | ICP 13828 | 48.99 | gh | ICP 10889 | 13.43 | g |
| ICP 12041 | 9.19 | f | ICP 11485 | 29.96 | d | ICP 10889 | 47.18 | h | ICP 13316 | 13.10 | g |
