# Supplementary material for: Genome-Wide DArTSeq Genotyping and Phenotypic Based Assessment of Within and Among Accessions Diversity and Effective Sample Size in the Diverse Sorghum, Pearl Millet, and Pigeonpea Landraces
Source: Front Plant Sci. 2020 Dec 14;11:587426. doi: 10.3389/fpls.2020.587426 (PMC7768014; doi:10.3389/fpls.2020.587426)
Supplement: Supplementary Figure 1 — Cluster dendrogram with unbiased bootstrap probability values for edges, with ward.D2 clustering for Gower's distances, for single plant phenotypic data (A) The cluster dendrogram of sorghum, (B) the cluster dendrogram of pigeonpea, and (C) Cluster dendrogram of pearl millet. [file Data_Sheet_1.zip › Supplemantary material_corrected/Table S9.docx]

**Table S9. Bootstrapping (Jaccard coefficients) values of clusters for DArTSeq SNP data**

| Cluster No. | Sorghum | Cluster No. | Pigeonpea | Cluster No. | Pearl millet |
| --- | --- | --- | --- | --- | --- |
| 12 | 0.429 | 20 | 0.479 | 34 | 0.369 |
| 19 | 0.636 | 10 | 0.496 | 27 | 0.391 |
| 20 | 0.675 | 15 | 0.547 | 14 | 0.426 |
| 34 | 0.721 | 3 | 0.568 | 4 | 0.502 |
| 28 | 0.738 | 4 | 0.570 | 31 | 0.521 |
| 23 | 0.855 | 16 | 0.607 | 1 | 0.542 |
| 3 | 0.951 | 21 | 0.624 | 8 | 0.563 |
| 32 | 0.958 | 17 | 0.628 | 9 | 0.566 |
| 15 | 0.963 | 2 | 0.644 | 3 | 0.577 |
| 1 | 0.968 | 8 | 0.647 | 7 | 0.589 |
| 30 | 0.973 | 27 | 0.659 | 13 | 0.633 |
| 25 | 0.977 | 5 | 0.672 | 30 | 0.664 |
| 33 | 0.983 | 1 | 0.764 | 6 | 0.671 |
| 17 | 0.995 | 24 | 0.770 | 21 | 0.672 |
| 36 | 0.997 | 31 | 0.773 | 18 | 0.675 |
| 2 | 1.000 | 26 | 0.790 | 35 | 0.693 |
| 4 | 1.000 | 7 | 0.793 | 26 | 0.699 |
| 5 | 1.000 | 36 | 0.810 | 12 | 0.705 |
| 6 | 1.000 | 30 | 0.820 | 32 | 0.746 |
| 7 | 1.000 | 19 | 0.837 | 19 | 0.766 |
| 8 | 1.000 | 13 | 0.840 | 23 | 0.771 |
| 9 | 1.000 | 14 | 0.845 | 25 | 0.782 |
| 10 | 1.000 | 29 | 0.888 | 24 | 0.784 |
| 11 | 1.000 | 6 | 0.891 | 10 | 0.785 |
| 13 | 1.000 | 22 | 0.908 | 15 | 0.800 |
| 14 | 1.000 | 28 | 0.924 | 2 | 0.802 |
| 16 | 1.000 | 25 | 0.956 | 5 | 0.816 |
| 18 | 1.000 | 23 | 0.958 | 17 | 0.821 |
| 21 | 1.000 | 18 | 0.965 | 11 | 0.827 |
| 22 | 1.000 | 11 | 0.971 | 29 | 0.853 |
| 24 | 1.000 | 33 | 0.972 | 28 | 0.864 |
| 26 | 1.000 | 9 | 0.982 | 16 | 0.917 |
| 27 | 1.000 | 34 | 0.985 | 22 | 0.921 |
| 29 | 1.000 | 35 | 0.987 | 33 | 0.926 |
| 31 | 1.000 | 12 | 1.000 | 20 | 0.967 |
| 35 | 1.000 | 32 | 1.000 | 36 | 0.994 |
