# Supplementary material for: Genome-Wide DArTSeq Genotyping and Phenotypic Based Assessment of Within and Among Accessions Diversity and Effective Sample Size in the Diverse Sorghum, Pearl Millet, and Pigeonpea Landraces
Source: Front Plant Sci. 2020 Dec 14;11:587426. doi: 10.3389/fpls.2020.587426 (PMC7768014; doi:10.3389/fpls.2020.587426)
Supplement: Supplementary Figure 1 — Cluster dendrogram with unbiased bootstrap probability values for edges, with ward.D2 clustering for Gower's distances, for single plant phenotypic data (A) The cluster dendrogram of sorghum, (B) the cluster dendrogram of pigeonpea, and (C) Cluster dendrogram of pearl millet. [file Data_Sheet_1.zip › Supplemantary material_corrected/TableS1, S2, S3, S4.docx]

**Table S1. List of accessions of sorghum used for phenotypic and genotypic within accession diversity evaluation**

| **Accession Number** | **Race** | **Subsp** | **Biological status** | **Country Source** | **Region** |
| --- | --- | --- | --- | --- | --- |
| IS 1128 | Bicolor | *bicolor* | Traditional cultivar/Landrace | India | Asia |
| IS 2134 | Guinea-kafir | *bicolor* | Traditional cultivar/Landrace | Ethiopia | Africa |
| IS 2153 | Bicolor | *bicolor* | Traditional cultivar/Landrace | Nigeria | Africa |
| IS 2348 | Kafir-caudatum | *bicolor* | Traditional cultivar/Landrace | Indonesia | Asia |
| IS 3399 | Kafir | *bicolor* | Traditional cultivar/Landrace | Botswana | Africa |
| IS 8330 | Caudatum | *bicolor* | Traditional cultivar/Landrace | India | Asia |
| IS 10897 | Wild | *drummondii* | Wild | India | Asia |
| IS 11005 | Wild | *drummondii* | Wild | Ethiopia | Africa |
| IS 12919 | Durra-bicolor | *bicolor* | Traditional cultivar/Landrace | Guadeloupe | America |
| IS 12965 | Caudatum | *bicolor* | Traditional cultivar/Landrace | Cuba | America |
| IS 13065 | Durra-bicolor | *bicolor* | Traditional cultivar/Landrace | Australia | Australia |
| IS 13068 | Kafir-durra | *bicolor* | Traditional cultivar/Landrace | Australia | Australia |
| IS 13211 | Bicolor | *bicolor* | Traditional cultivar/Landrace | Spain | Europe |
| IS 13215 | Durra | *bicolor* | Traditional cultivar/Landrace | Guatemala | America |
| IS 14010 | Caudatum-bicolor | *bicolor* | Traditional cultivar/Landrace | South Africa | Africa |
| IS 14485 | Wild | *verticilliflorum* | Wild | Sudan | Africa |
| IS 18234 | Guinea-caudatum | *bicolor* | Traditional cultivar/Landrace | Bangladesh | Asia |
| IS 18833 | Wild | *verticilliflorum* | Wild | Malawi | Africa |
| IS 21858 | Guinea-caudatum | *bicolor* | Traditional cultivar/Landrace | Russian Federation | Europe |
| IS 22407 | Guinea-durra | *bicolor* | Traditional cultivar/Landrace | Sudan | Africa |
| IS 22428 | Wild | *drummondii* | Wild | Sudan | Africa |
| IS 22606 | Guinea | *bicolor* | Traditional cultivar/Landrace | Sri Lanka | Asia |
| IS 25476 | Caudatum | *bicolor* | Traditional cultivar/Landrace | Burundi | Africa |
| IS 27325 | Guinea-bicolor | *bicolor* | Traditional cultivar/Landrace | Burkina Faso | Africa |
| IS 29508 | Kafir | *bicolor* | Traditional cultivar/Landrace | Lesotho | Africa |
| IS 29605 | Kafir-bicolor | *bicolor* | Traditional cultivar/Landrace | Lesotho | Africa |
| IS 31637 | Durra-caudatum | *bicolor* | Traditional cultivar/Landrace | Rwanda | Africa |
| IS 32252 | Durra-caudatum | *bicolor* | Traditional cultivar/Landrace | Yemen | Asia |
| IS 32263 | Durra | *bicolor* | Traditional cultivar/Landrace | Yemen | Asia |
| IS 34283 | Guinea | *bicolor* | Traditional cultivar/Landrace | Zimbabwe | Africa |
| IS 35217 | Caudatum-bicolor | *bicolor* | Traditional cultivar/Landrace | Pakistan | Asia |
| IS 35474 | Guinea-kafir | *bicolor* | Traditional cultivar/Landrace | Namibia | Africa |
| IS 40031 | Guinea-caudatum | *bicolor* | Traditional cultivar/Landrace | Uganda | Africa |
| IS 40161 | Durra | *bicolor* | Traditional cultivar/Landrace | Mali | Africa |
| IS 40238 | Durra-bicolor | *bicolor* | Traditional cultivar/Landrace | India | Asia |
| IS 33844 | Durra | *bicolor* | Traditional cultivar/Landrace | India | Asia |

**Table S2. List of accessions of pigeonpea used for phenotypic and genotypic within accession diversity evaluation**

| **Accession Number** | **Genus** | **Species** | **Biological status** | **Country Source** | **Region** |
| --- | --- | --- | --- | --- | --- |
| ICP 2309 | *Cajanus* | *cajan* | Traditional cultivar/Landrace | Nepal | Asia |
| ICP 6399 | *Cajanus* | *cajan* | Traditional cultivar/Landrace | Peru | South America |
| ICP 7057 | *Cajanus* | *cajan* | Traditional cultivar/Landrace | India | Asia |
| ICP 7621 | *Cajanus* | *cajan* | Traditional cultivar/Landrace | Colombia | South America |
| ICP 9122 | *Cajanus* | *cajan* | Traditional cultivar/Landrace | Russian Federation | Asia |
| ICP 9124 | *Cajanus* | *cajan* | Traditional cultivar/Landrace | Venezuela | South America |
| ICP 9877 | *Cajanus* | *cajan* | Traditional cultivar/Landrace | Puerto Rico | North America |
| ICP 10880 | *Cajanus* | *cajan* | Traditional cultivar/Landrace | Philippines | Asia |
| ICP 10889 | *Cajanus* | *cajan* | Traditional cultivar/Landrace | Indonesia | Asia |
| ICP 11475 | *Cajanus* | *cajan* | Traditional cultivar/Landrace | Sri Lanka | Asia |
| ICP 11480 | *Cajanus* | *cajan* | Traditional cultivar/Landrace | Nigeria | Africa |
| ICP 11485 | *Cajanus* | *cajan* | Traditional cultivar/Landrace | Thailand | Asia |
| ICP 11491 | *Cajanus* | *cajan* | Traditional cultivar/Landrace | Myanmar | Asia |
| ICP 12041 | *Cajanus* | *cajan* | Traditional cultivar/Landrace | Tanzania | Africa |
| ICP 12840 | *Cajanus* | *cajan* | Traditional cultivar/Landrace | Mozambique | Africa |
| ICP 12189 | *Cajanus* | *cajan* | Traditional cultivar/Landrace | South Africa | Africa |
| ICP 12190 | *Cajanus* | *cajan* | Traditional cultivar/Landrace | Ghana | Africa |
| ICP 13316 | *Cajanus* | *cajan* | Traditional cultivar/Landrace | Rwanda | Africa |
| ICP 13415 | *Cajanus* | *cajan* | Traditional cultivar/Landrace | Malawi | Africa |
| ICP 13546 | *Cajanus* | *cajan* | Traditional cultivar/Landrace | Trinidad and Tobago | South America |
| ICP 13575 | *Cajanus* | *cajan* | Traditional cultivar/Landrace | Sierra Leone | Africa |
| ICP 13628 | *Cajanus* | *cajan* | Traditional cultivar/Landrace | Ethiopia | Africa |
| ICP 13889 | *Cajanus* | *cajan* | Traditional cultivar/Landrace | Dominican Republic | North America |
| ICP 13999 | *Cajanus* | *cajan* | Traditional cultivar/Landrace | Rwanda | Africa |
| ICP 14059 | *Cajanus* | *cajan* | Traditional cultivar/Landrace | Guyana | South America |
| ICP 14233 | *Cajanus* | *cajan* | Traditional cultivar/Landrace | Zambia | Africa |
| ICP 14296 | *Cajanus* | *cajan* | Traditional cultivar/Landrace | Italy | Europe |
| ICP 14388 | *Cajanus* | *cajan* | Traditional cultivar/Landrace | Central African Republic | Africa |
| ICP 15148 | *Cajanus* | *cajan* | Traditional cultivar/Landrace | Congo | Africa |
| ICP 16344 | *Cajanus* | *cajan* | Traditional cultivar/Landrace | Argentina | South America |
| ICP 7035 | *Cajanus* | *cajan* | Traditional cultivar/Landrace | India | Asia |
| ICP 9150 | *Cajanus* | *cajan* | Traditional cultivar/Landrace | Kenya | Africa |
| ICP 13545 | *Cajanus* | *cajan* | Traditional cultivar/Landrace | Antigua and Barbuda | North America |
| ICP 13828 | *Cajanus* | *cajan* | Traditional cultivar/Landrace | Grenada | North America |
| ICP 14169 | *Cajanus* | *cajan* | Traditional cultivar/Landrace | Jamaica | North America |
| ICP 15122 | *Cajanus* | *cajan* | Traditional cultivar/Landrace | Uganda | Africa |

**Table S3. List of accessions of pearl millet used for phenotypic and genotypic within accession diversity evaluation**

| **Accession Number** | **Genus** | **Species** | **Biological status** | **Country Source** | **Region** |
| --- | --- | --- | --- | --- | --- |
| IP 3269 | *Pennisetum* | *glaucum* | Traditional cultivar/Landrace | India | Asia |
| IP 3389 | *Pennisetum* | *glaucum* | Traditional cultivar/Landrace | India | Asia |
| IP 3616 | *Pennisetum* | *glaucum* | Traditional cultivar/Landrace | India | Asia |
| IP 4952 | *Pennisetum* | *glaucum* | Traditional cultivar/Landrace | Uganda | Africa |
| IP 5253 | *Pennisetum* | *glaucum* | Traditional cultivar/Landrace | Niger | Africa |
| IP 5441 | *Pennisetum* | *glaucum* | Traditional cultivar/Landrace | Niger | Africa |
| IP 5900 | *Pennisetum* | *glaucum* | Traditional cultivar/Landrace | Senegal | Africa |
| IP 6037 | *Pennisetum* | *glaucum* | Traditional cultivar/Landrace | Central African Republic | Africa |
| IP 6109 | *Pennisetum* | *glaucum* | Traditional cultivar/Landrace | Niger | Africa |
| IP 6244 | *Pennisetum* | *glaucum* | Traditional cultivar/Landrace | Cameroon | Africa |
| IP 6434 | *Pennisetum* | *glaucum* | Traditional cultivar/Landrace | Mali | Africa |
| IP 7468 | *Pennisetum* | *glaucum* | Traditional cultivar/Landrace | Tanzania | Africa |
| IP 8761 | *Pennisetum* | *glaucum* | Traditional cultivar/Landrace | Botswana | Africa |
| IP 9446 | *Pennisetum* | *glaucum* | Traditional cultivar/Landrace | Ghana | Africa |
| IP 9824 | *Pennisetum* | *glaucum* | Traditional cultivar/Landrace | Mozambique | Africa |
| IP 10085 | *Pennisetum* | *glaucum* | Traditional cultivar/Landrace | Mali | Africa |
| IP 10471 | *Pennisetum* | *glaucum* | Traditional cultivar/Landrace | Zimbabwe | Africa |
| IP 10705 | *Pennisetum* | *glaucum* | Traditional cultivar/Landrace | Mali | Africa |
| IP 11577 | *Pennisetum* | *glaucum* | Traditional cultivar/Landrace | Burkina Faso | Africa |
| IP 11677 | *Pennisetum* | *glaucum* | Traditional cultivar/Landrace | Sudan | Africa |
| IP 11984 | *Pennisetum* | *glaucum* | Traditional cultivar/Landrace | Nigeria | Africa |
| IP 12138 | *Pennisetum* | *glaucum* | Traditional cultivar/Landrace | Nigeria | Africa |
| IP 12155 | *Pennisetum* | *glaucum* | Traditional cultivar/Landrace | Nigeria | Africa |
| IP 13112 | *Pennisetum* | *glaucum* | Traditional cultivar/Landrace | Niger | Africa |
| IP 13363 | *Pennisetum* | *glaucum* | Traditional cultivar/Landrace | Tanzania | Africa |
| IP 13459 | *Pennisetum* | *glaucum* | Traditional cultivar/Landrace | India | Asia |
| IP 14418 | *Pennisetum* | *glaucum* | Traditional cultivar/Landrace | Cameroon | Africa |
| IP 17632 | *Pennisetum* | *glaucum* | Traditional cultivar/Landrace | Togo | Africa |
| IP 18147 | *Pennisetum* | *glaucum* | Traditional cultivar/Landrace | Pakistan | Asia |
| IP 18157 | *Pennisetum* | *glaucum* | Traditional cultivar/Landrace | Mali | Africa |
| IP 19434 | *Pennisetum* | *glaucum* | Traditional cultivar/Landrace | Namibia | Africa |
| IP 20349 | *Pennisetum* | *glaucum* | Traditional cultivar/Landrace | Yemen | Asia |
| IP 20407 | *Pennisetum* | *glaucum* | Traditional cultivar/Landrace | Nigeria | Africa |
| IP 21640 | *Pennisetum* | *violaceum* | Wild | Niger | Africa |
| IP 21752 | *Pennisetum* | *mollissimum* | Wild | Niger | Africa |
| IP 22039 | *Pennisetum* | *violaceum* | Wild | Mali | Africa |

**Table S4. List of the quantitative and qualitative traits recorded in sorghum, pearl millet and pigeonpea**

| **Quantitative traits** | | |
| --- | --- | --- |
| **Sorghum** | **Pigeon pea** | **Pearl millet** |
| Days to flowering | Leaf length (cm) | Days to flowering |
| Days to maturity | Leaflet width (cm) | Number of leaves |
| Plant height (cm) | Plant height (cm) | Leaf length (cm) |
| Leaf blade length (cm) | Primary branches per plant | Leaf width (cm) |
| Leaf blade width (cm) | Secondary branches per plant | Plant height (cm) |
| Panicle excersion (cm) | Tertiary branches per plant | Basal tillers |
| Panicle length (cm) | Days to flowering | Stem thickness (cm) |
| Panicle width (cm) | Racemes per plant | Panicle length (cm) |
| Single plant yield (g) | Days to 75 percent maturity | Panicle width (cm) |
| Single plant seed yield (g) | Pod bearing length (cm) | **-** |
| **-** | Pod length (cm) | **-** |
| **-** | Pods per plant | **-** |
| **-** | Seeds per pod | **-** |
| **-** | 100 seed weight (g) | **-** |
| **-** | Seed yield per plant (g) | **-** |
| **-** | Dry plant weight (g) | **-** |
| **-** | Shelling percentage | **-** |
| **-** | Harvest index | **-** |
| **Qualitative traits** | | |
| **Sorghum** | **Pigeon pea** | **Pearl millet** |
| Panicle shape and compactness | Growth habitat | Bristle length |
| Glume colour | Plant Vigour | Seed shape |
| Glume covering | Stem thickness | Seed apex |
| Threshability | Plant pigmentation | Seed colour |
| Race | Leaflet shape | Endosperm texture |
| Seed colour | Leaf hairiness | Leaf sheath pigmentation |
| Seed lusture | Flowering pattern | Leaf blade pigmentation |
| Endosperm texture | Flower colour | Node pigmentation |
| Seed sub coat | Streak colour | Internode pigmentation |
| Mid rib colour | Streak pattern | Leaf sheath pubescence |
| Plant pigmentation | Pod colour | Node pubescence |
| Basal tillers | Pod shape | - |
| Nodal tillers | Pod hairiness | - |
| **-** | Leaf colour | **-** |
| **-** | Seed colour pattern | **-** |
| **-** | Primary seed colour | **-** |
| **-** | Secondary seed colour | **-** |
| **-** | Seed eye colour | **-** |
| **-** | Seed colour eye width | **-** |
| **-** | Seed shape | **-** |
| **-** | Seed hilum | **-** |
