# Supplementary material for: Genome-Wide DArTSeq Genotyping and Phenotypic Based Assessment of Within and Among Accessions Diversity and Effective Sample Size in the Diverse Sorghum, Pearl Millet, and Pigeonpea Landraces
Source: Front Plant Sci. 2020 Dec 14;11:587426. doi: 10.3389/fpls.2020.587426 (PMC7768014; doi:10.3389/fpls.2020.587426)
Supplement: Supplementary Figure 1 — Cluster dendrogram with unbiased bootstrap probability values for edges, with ward.D2 clustering for Gower's distances, for single plant phenotypic data (A) The cluster dendrogram of sorghum, (B) the cluster dendrogram of pigeonpea, and (C) Cluster dendrogram of pearl millet. [file Data_Sheet_1.zip › Supplemantary material_corrected/Table S11.docx]

| Sorghum | | Pigeonpea | | Pearl millet | |
| --- | --- | --- | --- | --- | --- |
| Accession number | Number of rare alleles | Accession number | Number of rare alleles | Accession number | Number of rare alleles |
| IS 14010 | 345 | ICP 14169 | 231 | IP 5253 | 3444 |
| IS 33844 | 378 | ICP 9124 | 239 | IP 18147 | 3462 |
| IS 40238 | 378 | ICP 9150 | 243 | IP 19434 | 3521 |
| IS 22428 | 380 | ICP 7035 | 263 | IP 18157 | 3612 |
| IS 27325 | 393 | ICP 13828 | 264 | IP 13363 | 3627 |
| IS 22606 | 418 | ICP 12190 | 267 | IP 12155 | 3811 |
| IS 25476 | 419 | ICP 9877 | 271 | IP 9446 | 3908 |
| IS 35217 | 453 | ICP 2309 | 272 | IP 9824 | 3947 |
| IS 13215 | 459 | ICP 14059 | 299 | IP 6434 | 4068 |
| IS 13068 | 463 | ICP 10889 | 310 | IP 4952 | 4129 |
| IS 29508 | 491 | ICP 13628 | 316 | IP 10705 | 4179 |
| IS 1128 | 492 | ICP 14296 | 319 | IP 11577 | 4229 |
| IS 18234 | 506 | ICP 13415 | 321 | IP 11984 | 4253 |
| IS 40161 | 507 | ICP 11480 | 325 | IP 20407 | 4312 |
| IS 13065 | 510 | ICP 11491 | 335 | IP 10085 | 4336 |
| IS 11005 | 518 | ICP 13546 | 350 | IP 3389 | 4362 |
| IS 35474 | 537 | ICP 12189 | 352 | IP 10471 | 4481 |
| IS 29605 | 543 | ICP 12840 | 362 | IP 3269 | 4537 |
| IS 31637 | 551 | ICP 15122 | 364 | IP 12138 | 4587 |
| IS 32263 | 552 | ICP 9122 | 384 | IP 13112 | 4686 |
| IS 12965 | 565 | ICP 13999 | 391 | IP 8761 | 4854 |
| IS 22407 | 583 | ICP 6399 | 392 | IP 6037 | 5003 |
| IS 13211 | 604 | ICP 16344 | 419 | IP 6244 | 5044 |
| IS 2134 | 627 | ICP 11475 | 435 | IP 11677 | 5051 |
| IS 12919 | 678 | ICP 7621 | 439 | IP 21752 | 5095 |
| IS 40031 | 680 | ICP 15148 | 453 | IP 14418 | 5315 |
| IS 3399 | 744 | ICP 7057 | 475 | IP 3616 | 5376 |
| IS 34283 | 776 | ICP 12041 | 502 | IP 5900 | 5422 |
| IS 2348 | 894 | ICP 14388 | 555 | IP 5441 | 5571 |
| IS 8330 | 919 | ICP 13575 | 576 | IP 17632 | 5638 |
| IS 10897 | 1057 | ICP 14233 | 684 | IP 13459 | 5789 |
| IS 2153 | 1190 | ICP 13316 | 688 | IP 6109 | 5858 |
| IS 14485 | 1347 | ICP 13545 | 711 | IP 20349 | 6266 |
| IS 18833 | 1770 | ICP 10880 | 752 | IP 22039 | 6458 |
| IS 32252 | 1932 | ICP 11485 | 845 | IP 7468 | 6488 |
| IS 21858 | 3057 | ICP 13889 | 878 | IP 21640 | 6726 |

Table S11. Number of rare alleles preserved in the recommended sample size estimated from DArTSeq-SNP data of sorghum, pigeonpea, and pearl millet.
