# Supplementary material for: Genome-Wide DArTSeq Genotyping and Phenotypic Based Assessment of Within and Among Accessions Diversity and Effective Sample Size in the Diverse Sorghum, Pearl Millet, and Pigeonpea Landraces
Source: Front Plant Sci. 2020 Dec 14;11:587426. doi: 10.3389/fpls.2020.587426 (PMC7768014; doi:10.3389/fpls.2020.587426)
Supplement: Supplementary Figure 1 — Cluster dendrogram with unbiased bootstrap probability values for edges, with ward.D2 clustering for Gower's distances, for single plant phenotypic data (A) The cluster dendrogram of sorghum, (B) the cluster dendrogram of pigeonpea, and (C) Cluster dendrogram of pearl millet. [file Data_Sheet_1.zip › Supplemantary material_corrected/Table S10.docx]

**Table S10.** Probability of attribution of all the single plants in each accession into different groups based on the discriminant analysis of principle components on DArTSeq – SNP data (K=36)

|  | Probability of attribution (a-scores) | | |
| --- | --- | --- | --- |
| Cluster | Sorghum | Pigeonpea | Pearl millet |
| 1 | 0.91 | 0.82 | 0.40 |
| 2 | 0.80 | 0.82 | 0.77 |
| 3 | 0.93 | 0.52 | 0.15 |
| 4 | 0.83 | 0.78 | 0.81 |
| 5 | 0.83 | 0.73 | 0.76 |
| 6 | 0.83 | 0.75 | 0.79 |
| 7 | 0.87 | 0.71 | 0.50 |
| 8 | 0.93 | 0.35 | 0.78 |
| 9 | 0.80 | 0.79 | 0.23 |
| 10 | 0.89 | 0.88 | 0.79 |
| 11 | 0.84 | 0.85 | 0.33 |
| 12 | 0.79 | 0.91 | 0.78 |
| 13 | 0.83 | 0.83 | 0.68 |
| 14 | 0.90 | 0.85 | 0.51 |
| 15 | 0.95 | 0.82 | 0.80 |
| 16 | 0.79 | 0.83 | 0.74 |
| 17 | 0.87 | 0.81 | 0.79 |
| 18 | 0.91 | 0.77 | 0.71 |
| 19 | 0.97 | 0.68 | 0.77 |
| 20 | 0.36 | 0.72 | 0.79 |
| 21 | 0.88 | 0.83 | 0.74 |
| 22 | 0.90 | 0.81 | 0.57 |
| 23 | 0.89 | 0.75 | 0.57 |
| 24 | 0.85 | 0.81 | 0.82 |
| 25 | 0.47 | 0.81 | 0.69 |
| 26 | 0.79 | 0.74 | 0.74 |
| 27 | 0.84 | 0.60 | 0.72 |
| 28 | 0.87 | 0.62 | 0.72 |
| 29 | 0.81 | 0.86 | 0.72 |
| 30 | 0.88 | 0.78 | 0.85 |
| 31 | 0.70 | 0.71 | 0.63 |
| 32 | 0.13 | 0.86 | 0.35 |
| 33 | 0.87 | 0.89 | 0.60 |
| 34 | 0.85 | 0.70 | 0.61 |
| 35 | 0.93 | 0.05 | 0.63 |
| 36 | 0.87 | 0.79 | 0.64 |
