# Supplementary material for: Genome-Wide DArTSeq Genotyping and Phenotypic Based Assessment of Within and Among Accessions Diversity and Effective Sample Size in the Diverse Sorghum, Pearl Millet, and Pigeonpea Landraces
Source: Front Plant Sci. 2020 Dec 14;11:587426. doi: 10.3389/fpls.2020.587426 (PMC7768014; doi:10.3389/fpls.2020.587426)
Supplement: Supplementary Figure 1 — Cluster dendrogram with unbiased bootstrap probability values for edges, with ward.D2 clustering for Gower's distances, for single plant phenotypic data (A) The cluster dendrogram of sorghum, (B) the cluster dendrogram of pigeonpea, and (C) Cluster dendrogram of pearl millet. [file Data_Sheet_1.zip › Supplemantary material_corrected/Table S6.docx]

**Table 6. Levene’s test for significant differences in population variances**

| **Sorghum** | | | | | |
| --- | --- | --- | --- | --- | --- |
|  | Df | | Df-residual | F-value | Pr(>F) |
| Days to flowering | 35 | | 6382 | 25.03 | 3.17E-150*** |
| Days to maturity | 35 | | 6126 | 6.32 | 2.57E-28*** |
| Plant height (cm) | 35 | | 6301 | 29.72 | 5.86E-180*** |
| Leaf blade length (cm) | 35 | | 6301 | 11.41 | 3.64E-61*** |
| Leaf blade width (cm) | 35 | | 6301 | 11.48 | 1.18E-61*** |
| Panicle excersion (cm) | 35 | | 6301 | 19.97 | 2.42E-117*** |
| Panicle length (cm) | 35 | | 6301 | 4.18 | 2.29E-15*** |
| Panicle width (cm) | 35 | | 6301 | 65.48 | 0*** |
| Single plant yield (g) | 35 | | 6125 | 3.26 | 3.21E-10*** |
| Single plant seed yield (g) | 35 | | 6125 | 28.94 | 1.18E-174*** |
| **Pigeonpea** | | | | | |
|  | Df | | Df-residual | F-value | Pr(>F) |
| Leaf length (cm) | 35 | | 1079 | 6.25 | 2.16E-25*** |
| Leaflet width (cm) | 35 | | 1079 | 5.46 | 3.62E-21*** |
| Plant height (cm) | 35 | | 1079 | 16.88 | 3.21E-79*** |
| Primary branches per plant | 35 | | 1079 | 12.52 | 3.55E-58*** |
| Secondary branches per plant | 35 | | 1079 | 8.63 | 3.97E-38*** |
| Tertiary branches per plant | 35 | | 1079 | 6.01 | 3.91E-24*** |
| Days to flowering | 35 | | 1079 | 22.62 | 1.47E-104*** |
| Racemes per plant | 35 | | 1079 | 6.85 | 1.31E-28*** |
| Days to 75 percent maturity | 35 | | 1079 | 14.97 | 3.53E-70*** |
| Pod bearing length (cm) | 35 | 1079 | | 12.58 | 1.64E-58*** |
| Pod length (cm) | 35 | | 1079 | 10.88 | 6.74E-50*** |
| Pods per plant | 35 | | 1079 | 10.84 | 1.16E-49*** |
| Seeds per pod | 35 | | 1079 | 6.53 | 6.16E-27*** |
| 100 seed weight (g) | 35 | | 1079 | 17.14 | 2.17E-80*** |
| Seed yield per plant (g) | 35 | | 1079 | 6.42 | 2.48E-26*** |
| Dry plant weight (g) | 35 | | 1079 | 8.63 | 3.80E-38*** |
| Shelling percentage | 35 | | 1079 | 9.26 | 1.95E-41*** |
| Harvest index | 35 | | 1079 | 6.89 | 7.95E-29*** |
| **Peral millet** | | | | | |
|  | Df | | Df-residual | F-value | Pr(>F) |
| Days to flowering | 35 | | 3093 | 4.16 | 5.05E-15*** |
| Number of leaves | 35 | | 3527 | 2.87 | 4.00E-08*** |
| Leaf length (cm) | 35 | | 3527 | 3.77 | 7.25E-13*** |
| Leaf width (cm) | 35 | | 3527 | 5.64 | 1.01E-23*** |
| Plant height (cm) | 35 | | 3475 | 6.41 | 2.25E-28*** |
| Basal tillers | 35 | | 3475 | 47.23 | 6.82E-263*** |
| Stem thickness (cm) | 35 | | 3475 | 4.43 | 1.44E-16*** |
| Panicle length (cm) | 35 | | 3380 | 22.99 | 7.17E-130*** |
| Panicle width (cm) | 35 | | 3379 | 16.36 | 4.73E-90*** |

Significant codes:

*** - significant at 0.001

** - significant at 0.01

* - significant at 0.05
